# Multigenic phylogeny and analysis of tree incongruences in Triticeae (Poaceae)

**DOI:** 10.1186/1471-2148-11-181

**Published:** 2011-06-24

**Authors:** Juan S Escobar, Céline Scornavacca, Alberto Cenci, Claire Guilhaumon, Sylvain Santoni, Emmanuel JP Douzery, Vincent Ranwez, Sylvain Glémin, Jacques David

**Affiliations:** 1 Institut National de la Recherche Agronomique, Centre de Montpellier, UMR Diversité et Adaptation des Plantes Cultivées, Domaine de Melgueil, 34130 Mauguio, France; 2 Institut des Sciences de l'Evolution, UMR 5554 CNRS, Université Montpellier II, Place Eugène Bataillon, 34095 Montpellier Cedex 5, France; 3 Laboratoire d'Informatique, de Robotique et de Microélectronique de Montpellier, UMR 5506, Université Montpellier II, 161 rue Ada, 34392 Montpellier Cedex 5, France; 4 Montpellier Supagro, Centre International d'Etudes Supérieures en Sciences Agronomiques, UMR Diversité et Adaptation des Plantes Cultivées, 2 Place Pierre Viala, 34060 Montpellier Cedex 1, France; 5 Department of Ecology and Evolutionary Biology, University of Toronto, 25 Willcocks Street, M5S 3B2 Toronto, Canada; 6 University of Tübingen, Faculty of Computer Science, Chair of Algorithms in Bioinformatics, Sand 14, 72076 Tübingen, Germany; 7 Institut de Recherche pour le Développement, UMR RPB-Equipe DIVA, 911 Avenue Agropolis, 34394 Montpellier Cedex 5, France

## Abstract

**Background:**

Introgressive events (e.g., hybridization, gene flow, horizontal gene transfer) and incomplete lineage sorting of ancestral polymorphisms are a challenge for phylogenetic analyses since different genes may exhibit conflicting genealogical histories. Grasses of the Triticeae tribe provide a particularly striking example of incongruence among gene trees. Previous phylogenies, mostly inferred with one gene, are in conflict for several taxon positions. Therefore, obtaining a resolved picture of relationships among genera and species of this tribe has been a challenging task. Here, we obtain the most comprehensive molecular dataset to date in Triticeae, including one chloroplastic and 26 nuclear genes. We aim to test whether it is possible to infer phylogenetic relationships in the face of (potentially) large-scale introgressive events and/or incomplete lineage sorting; to identify parts of the evolutionary history that have not evolved in a tree-like manner; and to decipher the biological causes of gene-tree conflicts in this tribe.

**Results:**

We obtain resolved phylogenetic hypotheses using the supermatrix and Bayesian Concordance Factors (BCF) approaches despite numerous incongruences among gene trees. These phylogenies suggest the existence of 4-5 major clades within Triticeae, with *Psathyrostachys *and *Hordeum *being the deepest genera. In addition, we construct a multigenic network that highlights parts of the Triticeae history that have not evolved in a tree-like manner. *Dasypyrum, Heteranthelium *and genera of clade V, grouping *Secale, Taeniatherum, Triticum *and *Aegilops*, have evolved in a reticulated manner. Their relationships are thus better represented by the multigenic network than by the supermatrix or BCF trees. Noteworthy, we demonstrate that gene-tree incongruences increase with genetic distance and are greater in telomeric than centromeric genes. Together, our results suggest that recombination is the main factor decoupling gene trees from multigenic trees.

**Conclusions:**

Our study is the first to propose a comprehensive, multigenic phylogeny of Triticeae. It clarifies several aspects of the relationships among genera and species of this tribe, and pinpoints biological groups with likely reticulate evolution. Importantly, this study extends previous results obtained in *Drosophila *by demonstrating that recombination can exacerbate gene-tree conflicts in phylogenetic reconstructions.

## Background

When reconstructing the phylogeny of a biological group it is implicitly assumed that species split in a tree-like manner and that all characters (e.g., all genes in the genome) reveal the same genealogical history that has occurred in each lineage after the split from a common ancestor. When these two assumptions are met phylogenetic trees inferred from one or a few genes can be used as proxies of the species tree. However, recent studies have shown that trees inferred from different genes may conflict with each other and that violation of these assumptions is more common than previously thought [1-10].

Incongruence may appear among gene trees for various reasons. If the genes used to infer the phylogenetic relationships among genera and species are sampled from introgressed portions of the genome produced by hybridization, gene flow or horizontal gene transfer, the trees obtained likely reflect the history of the introgression rather than the history of lineage splitting [11,12]. The genealogical histories of individual genes may also be misleading due to retention and stochastic sorting of ancestral polymorphisms caused by incomplete lineage sorting. This is especially likely when the effective population size of a given lineage is large with respect to the time elapsed since divergence [13-15]. In this case, genetic drift is unlikely to have brought alleles to fixation before subsequent divergence [1,6]. Finally, gene duplication followed by gene loss may lead to incongruence because paralogous gene copies are incorrectly inferred to be orthologous [16].

Whatever their origin, incongruences among gene trees require careful attention for several reasons. First, they affect the interpretation of morphological and molecular patterns of evolution. Second, they maintain extensive instability in taxonomy. Third, they complicate the choice of wild taxa as sources of novel genes in breeding programs (e.g., genes conferring resistance to pathogens, tolerance to salt, low temperatures and drought). Finally, uncertainty in phylogenetic relationships may lead to inadequate conservation decisions (e.g., the protection of particular species or habitats).

In prokaryotes, some authors argue that numerous hybridizations and gene transfers preclude the possibility and the meaning of a tree-like representation of a species history [17,18]. In plants too it has been argued that, in some cases, reticulate evolution is more appropriate than a tree-like description [19]. On the contrary, other authors argue that despite incongruences it is possible to reconstruct phylogenies and tree-like histories [20,21]. Among angiosperms, Triticeae grasses provide a particularly striking example of incongruence among gene trees, suggesting reticulate evolution [22]. This tribe comprises species of major economic importance, including wheat, barley and rye. In recent years, attempts to try and sort out the phylogenetic details of the group, based on analyses of single-copy nuclear genes [23-26], highly repetitive nuclear DNA [27], internal transcribed spacers [28], and chloroplastic genes [29-31], failed to lead to any consensual definition of clades. Current evidence suggests that different portions of the nuclear and chloroplastic genomes have different genealogical histories. Because published trees are in conflict for almost all taxon positions we do not know whether the historical relationships among the genera and species of this tribe can be resolved, or not, in a tree-like manner and, if so, what are the real phylogenetic relationships. In this paper, we use the most comprehensive molecular dataset to date in Triticeae, including 27 gene fragments, with the aim to (i) reconstruct a multigenic phylogeny of this tribe, (ii) quantify tree incongruences, and (iii) explore possible factors affecting incongruence, including the frequency of recombination, chromosomal location and evolutionary rate.

## Methods

### Species Studied and Loci Sampled

Nineteen diploid species, spanning 13 genera of Triticeae, were analyzed. These species were selected because they belong to most phylogenetic clades recognized so far [22,26,29] and represent most of the diversity of diploid genera (68% according to [22] and [32]), life styles (annual and perennial), mating systems (self-compatible and self-incompatible), and geographic origin (Europe, Middle East, Asia, North America and Australia). One or two accessions per species were obtained from the United States Department of Agriculture (USDA), National Plant Germplasm System (available at http://www.ars-grin.gov/npgs/index.html), making a total of 32 accessions (Table 1). Although *Bromus *is supposed to be the closest outgroup of Triticeae [26,33,34], to simplify primer design we preferred to use *Brachypodium distachyon*, a more distant species for which the complete genome is available [35]. As the ingroup topology may depend upon the choice of a single outgroup, *Zea mays *and *Oryza sativa *were also incorporated as additional, more distant outgroups. The choice of distant outgroups may increase the number of homoplasies. However, owing to the selective constraints likely acting on the coding sequences we used (see below), it is likely they have been affected by low substitutional saturation and hence by a low homoplasy level.

**Table 1:** Species names, accession numbers in the USDA database, and geographic origin of sampled Triticeae

Species	Accession	Origin
*Aegilops longissima*	PI 330486	Unknown
*Aegilops longissima*	PI 604110	Israel
*Aegilops speltoides *var. *speltoides*	PI 449338	Israel
*Aegilops speltoides *var. *ligustica*	PI 560528	Turkey
*Aegilops tauschii*	PI 603233	Azerbaijan
*Aegilops tauschii*	PI 603254	Iran
*Agropyron mongolicum*	PI 499391	China
*Agropyron mongolicum*	PI 598482	Unknown
*Australopyrum retrofractum*	PI 531553	Australia
*Australopyrum retrofractum*	PI 533013	Australia
*Brachypodium *sp.*	PI 317418	Afghanistan
*Dasypyrum villosum*	PI 251477	Turkey
*Dasypyrum villosum*	PI 598396	Greece
*Eremopyrum bonaepartis*	PI 203442	Turkey
*Eremopyrum triticeum*	PI 502364	Russia
*Henrardia persica*	PI 401347	Iran
*Henrardia persica*	PI 577112	Turkey
*Heteranthelium piliferum*	PI 401354	Iran
*Hordeum bogdanii*	PI 499498	China
*Hordeum marinum *subsp. *marinum*	PI 401364	Iran
*Hordeum vulgare *subsp. *spontaneum*	PI 282582	Israel
*Hordeum vulgare *subsp. *spontaneum*	PI 282585	Israel
*Psathyrostachys juncea*	PI 314668	Former USSR
*Psathyrostachys juncea*	PI 75737	Former USSR
*Pseudoroegneria libanotica*	PI 228389	Iran
*Pseudoroegneria libanotica*	PI 401274	Iran
*Pseudoroegneria spicata*	PI 563870	United States
*Secale cereale*	PI 561793	Turkey
*Taeniatherum caput-medusae*	PI 577708	Turkey
*Taeniatherum caput-medusae*	PI 598389	Turkey
*Triticum monococcum *subsp. *aegilopoides*	PI 272519	Hungary
*Triticum monococcum *subsp. *aegilopoides*	PI 427990	Lebanon

*Species misidentified in the USDA database as *Eremopyrum triticeum*

Orthologous coding sequences (cDNA) of one gene fragment from the chloroplast (*MATK*) and 26 nuclear gene fragments located on three different chromosomes (out of the seven chromosomes representative of Triticeae) were sequenced for each accession (Table 2; GenBank: HM539308-HM540073). Sequences of *B. distachyon *were obtained from the US Department of Energy Joint Genome Institute http://www.jgi.doe.gov/. Sequences of *Z. mays *and *O. sativa *were obtained from the GenBank.

**Table 2:** Relevant phylogenetic and genomic parameters for sequenced loci

Locus	Alignment length (bp)	Genomic location	Relative distance to the centromere	Evolutionary rate	Shape parameter α	Proportion of variable sites	TD supermatrix	TDBUCKy
LOC_Os01g01790	860	Chr. 3S, Tel.	0.976	1.687	0.527	0.313	0.152	0.246
LOC_Os01g09300	861	Chr. 3S, Tel.	0.722	0.883	0.342	0.285	0.207	0.272
LOC_Os01g11070	1050	Chr. 3S, Cen.	0.652	1.033	0.380	0.305	0.299	0.264
LOC_Os01g13200	897	Chr. 3S, Cen.	0.568	0.659	0.270	0.220	0.140	0.173
LOC_Os01g19470	942	Chr. 3S, Cen.	0.352	0.906	0.687	0.321	0.104	0.109
LOC_Os01g21160	1017	Chr. 3S, Cen.	0.307	1.596	0.475	0.393	0.236	0.291
LOC_Os01g24680	1014	Chr. 3S, Cen.	0.184	0.875	0.805	0.260	0.371	0.395
LOC_Os01g37560	1005	Chr. 3L, Cen.	0.160	1.060	0.392	0.310	0.158	0.201
LOC_Os01g39310	945	Chr. 3L, Cen.	0.202	0.989	0.328	0.290	0.108	0.243
LOC_Os01g48720	939	Chr. 3L, Cen.	0.417	1.252	0.805	0.399	0.203	0.288
LOC_Os01g53720	1101	Chr. 3L, Cen.	0.526	0.921	0.521	0.320	0.170	0.170
LOC_Os01g55530	1068	Chr. 3L, Cen.	0.567	0.890	0.426	0.309	0.131	0.074
LOC_Os01g56630	915	Chr. 3L, Cen.	0.592	0.731	0.504	0.312	0.105	0.163
LOC_Os01g60230	999	Chr. 3L, Cen.	0.673	0.929	0.355	0.283	0.202	0.159
LOC_Os01g61720	935	Chr. 3L, Tel.	0.705	1.131	0.385	0.328	0.080	0.098
LOC_Os01g62900	951	Chr. 3L, Tel.	0.732	0.897	0.241	0.257	0.113	0.290
LOC_Os01g67220	1101	Chr. 3L, Tel.	0.827	1.303	0.414	0.322	0.238	0.245
LOC_Os01g68770	998	Chr. 3L, Tel.	0.862	1.307	0.631	0.278	0.287	0.272
LOC_Os01g70670	883	Chr. 3L, Tel.	0.898	0.899	0.404	0.310	0.105	0.210
LOC_Os01g72220	1131	Chr. 3L, Tel.	0.933	0.974	0.253	0.255	0.279	0.385
LOC_Os01g73790	966	Chr. 3L, Tel.	0.965	0.850	0.689	0.180	0.227	0.244
eIFiso4E	630	Chr. 1L, Cen.	NA	0.952	1.004	0.128	0.221	0.430
CRTISO	529	Chr. 4L	NA	1.165	0.807	0.163	0.347	0.329
PinA	456	Chr. 5S	NA	1.375	0.243	0.189	0.506	0.382
PinB	453	Chr. 5S	NA	2.411	0.264	0.218	0.297	0.303
PSY2	461	NA	NA	0.978	0.648	0.150	0.246	0.372
MATK	1545	Chloroplast	NA	0.462	0.373	0.177	0.128	0.217

Chr.: chromosome; S: short arm; L: long arm; Tel.: telomere; Cen.: centromere; TD: triplet distance (relative to the supermatrix or BUCKy trees); NA: not available.

### RNA Extraction, cDNA Synthesis, PCR Amplification and Sequencing

Total RNA was extracted from 100 mg of young leaves using the RNeasy Plant Mini Kit (Qiagen). cDNA was synthesized from this RNA using oligo-dT primers with the Reverse Transcription System kit (Promega), following the manufacturer's protocol. For each gene fragment a couple of primers were designed on conserved regions identified based on alignments of barley and wheat EST (Additional file 1, Table S1). PCR amplification was performed on cDNA and amplification products were purified with the AMPure kit (Agencourt). Sanger sequencing was performed on amplicons with the same primers used in the PCR amplification. The BigDye Terminator v3.1 Cycle Sequencing Kit (Applied BioSystems) was used with 1.0 μl of BidDye v3.1 enzymatic reaction mix. The reactions were purified with the CleanSEQ kit (Agencourt) and separated on a 3130×l Genetic Analyzer (Applied BioSystems).

### Orthology Determination and Location of Loci on the Triticeae Genome

Because no genome of Triticeae species has been sequenced yet the orthology of the 27 sequenced gene fragments was established indirectly. Two observations strongly suggest the uniqueness of our sequences in the genome. First, all the sequenced loci are present in single copy in rice and *B. distachyon*, two species whose complete genomes are available. Second, according to EST data, they are expressed in one copy in diploid barley and in three copies in hexaploid wheat, which suggests the existence of a single copy per genome. The probability that our sequences coexist with paralogs is therefore limited.

From the 26 sequenced nuclear loci, 21 were derived from the rice chromosome 1, known to be collinear with the wheat chromosome 3B [36-38]. We assumed that chromosomal locations are mostly conserved across Triticeae [36-40] and used the location of rice orthologs as a proxy of their chromosomal position. Moreover, we checked that using *B. distachyon *as reference did not alter the estimated physical position. The relative distance to the centromere of each gene fragment was then computed assuming that the chromosome fraction separating them from the centromere was proportional in rice chromosome 1 and Triticeae chromosome 3 [36,38]. The centromere is located around 17 Mb from the telomere of the short arm in rice and 388 Mb in wheat [36,41].

Wheat has a strong recombination gradient [41,42], like other Triticeae species [39,43,44], which fits a positive exponential function from centromeres to telomeres [45]. The 21 loci located on chromosome 3 were thus suitable candidates to study the impact of recombination intensity in gene evolution. These loci were classified as centromeric (physical distance < 70% of chromosome arm), for which recombination is low, and telomeric (physical distance > 70% of chromosome arm), which concentrate most recombination events [45]. Due to the strong non-linear relationship between the physical and genetic map, the genetic distance along the chromosome was approximated according to reference [45]. Akhunov et al. [45] estimated the centimorgan per mega base (cM/Mb) ratio as a function of the percent of chromosome arm. To obtain the genetic distance in cM, we integrated the equation given in figure 1 in [45] and multiplied the result by the arm length:(1)

where *L *is the length of the chromosome arm (388 Mb for the short arm and 437 Mb for the long arm; [41]) and *x *is the relative distance to the centromere. To follow the evolution of recombination along the chromosome, positive (respectively negative) distances were assigned to the long (respectively short) chromosome arm.

In addition to the 21 nuclear loci located on chromosome 3, two loci corresponding to the hardness gene (*PinA *and *PinB*; [46]), one gene fragment corresponding to a eukaryotic initiation factor involved in translational regulation (*eIFiso4E*), and two gene fragments involved in the carotenoid biosynthetic pathway (*CRTISO *and *PSY2*; [36,47]), were sequenced. Positions of loci *PinA *and *PinB *were obtained from published data [46]. Positions of *eIFiso4E *and *CRTISO *were inferred from synteny with rice. The position of *PSY2 *is undetermined.

### Individual Gene Trees

Raw sequence data were aligned with the Staden Package [48] and the resulting alignments were manually corrected. Alignments for individual loci were analyzed using maximum likelihood (ML) and Bayesian approaches. ML analyses were conducted using the best-fitting model of sequence evolution, based on Akaike Information Criterion (AIC) using ModelTest 3.7 [49] (Table 3). PAUP* 4.0b10 [50] was used to obtain the highest-likelihood phylogenetic trees (heuristic search with neighbor-joining starting tree, tree bisection-reconnection branch swapping and 100 bootstrap replicates). Bayesian analyses were performed with MrBayes 3.1.2 [51,52] with the following priors: Dirichlet priors (1,1,1,1) for base frequencies and (1,1,1,1,1) for General Time Reversible (GTR) parameters scaled to the G-T rate, a uniform (0.05,50) and (0,1) priors for the gamma (Γ) shape and the proportion of invariable sites (I), and an exponential (10.0) prior for branch lengths. Metropolis-coupled Markov Chain Monte Carlo analyses (MCMCMC) were run with random starting trees and five simultaneous, sequentially heated independent chains. Analyses were run for 10,000,000 generations. We used the BPCOMP program implemented in PhyloBayes 2.3c [53] to determine appropriate convergence of the chains (i.e., the maximum difference [*maxdiff*] between posterior probabilities attached to the same clade as evidenced by independent chains is < 0.10). A burn-in was discarded after identifying the stationary phase.

**Table 3:** Best-fitting model of sequence evolution for each locus

Locus	Model	-lnL	AIC	α	I
LOC_Os01g01790	TrN + Γ + I	3,812.85	7,747.70	0.527	0.470
LOC_Os01g09300	K80 + Γ	3,202.24	6,520.48	0.342	-
LOC_Os01g11070	HKY + Γ	4,079.53	8,295.06	0.380	-
LOC_Os01g13200	HKY + Γ	2,884.00	5,900.00	0.270	-
LOC_Os01g19470	HKY + Γ	3,442.45	7,008.90	0.687	-
LOC_Os01g21160	TrN + Γ	5,012.34	10,136.68	0.475	-
LOC_Os01g24680	TrN + Γ + I	3,650.18	7,428.36	0.805	0.435
LOC_Os01g37560	HKY + Γ	3,696.29	7,486.58	0.392	-
LOC_Os01g39310	K80 + Γ	3,293.51	6,693.03	0.328	-
LOC_Os01g48720	HKY + Γ	4,172.17	8,478.34	0.805	-
LOC_Os01g53720	HKY + Γ	4,102.88	8,329.77	0.521	-
LOC_Os01g55530	K80 + Γ	4,250.90	8,635.80	0.426	-
LOC_Os01g56630	HKY + Γ	3,285.91	6,715.82	0.504	-
LOC_Os01g60230	HKY + Γ	3,514.88	7,111.76	0.355	-
LOC_Os01g61720	TrN + Γ	3,730.58	7,565.15	0.385	-
LOC_Os01g62900	TrN + Γ	3,344.32	6,830.64	0.241	-
LOC_Os01g67220	TrN + Γ	4,205.56	8,529.13	0.414	-
LOC_Os01g68770	TrN + Γ + I	4,137.81	8,397.62	0.631	0.462
LOC_Os01g70670	K80 + Γ	3,362.32	6,846.63	0.404	-
LOC_Os01g72220	K80 + Γ	3,721.70	7,559.39	0.253	-
LOC_Os01g73790	TrNef + Γ + I	2,953.10	6,028.21	0.689	0.617
eIFiso4E	TrN + Γ + I	1,592.24	3,300.49	1.004	0.666
CRTISO	TVM + Γ + I	1,515.13	3,162.26	0.807	0.621
PinA	K80 + Γ	1,345.73	2,779.45	0.243	-
PinB	GTR + Γ	1,460.81	2,993.62	0.264	-
PSY2	K80 + Γ + I	1,210.07	2,530.14	0.648	0.596
MATK	TVM + Γ	4,255.67	8,659.34	0.373	-
Supermatrix	GTR + I + Γ	63,591.59	127,037.19	0.799	0.580

Γ: gamma distribution; I: proportion of invariable sites; lnL = log-likelihood; AIC: Akaike Information Criterion; α: shape parameter of the gamma distribution.

### Multigenic Trees and Network

We obtained a multigenic, supermatrix tree by concatenating alignments of all 27 loci (24,652 bp). ML analysis of this supermatrix was performed in the same way as individual locus alignments using a GTR+Γ+I model. Bayesian inference was performed by partitioning the concatenate alignment on the basis of individual loci using a GTR+Γ+I model. Chains were run for 10,000,000 generations.

Multigenic, Bayesian Concordance Factors (BCF) were estimated using BUCKy 1.3.1 [54]. BCF estimates the degree of conflict of individual gene trees and accounts for all biological processes resulting in different phylogenies (e.g., introgression, incomplete lineage sorting). We summarized information from five MCMCMC chains obtained in individual locus analyses with MrBayes and removed 50% of the samples from each chain as burn-in. Then, BCF were estimated using six a priori levels of discordance among loci (α = 0.1, 0.5, 1, 5, 10 and 100) and 1,000,000 generations in each run.

Supermatrix and BCF analyses provide powerful means of using the evidence from all characters in the final estimation of the phylogenetic tree [55]. However, they implicitly assume that species split in a tree-like manner, which would not be the case when hybridization and/or lineage sorting have played an important role in the history of a group, as seems to be the case in Triticeae. To identify regions of the phylogeny of Triticeae that have not evolved in a tree-like manner, we constructed a multigenic network summarizing information conveyed by individual gene trees. The 27 gene trees were modified using the PhySIC_IST preprocess of source trees [56]. This preprocess aims at reducing source tree conflicts by eliminating a topological resolution when it is significantly less frequent in source trees than an alternative conflicting resolution. We applied a correction threshold of 0.9 to only keep strongly supported incongruences. Then, a network displaying all clades present in at least one among the modified gene trees was computed using the Cass algorithm [57] implemented in Dendroscope 2 [58], inputted with the Z-closure of the modified trees [59].

### Incongruence Quantification

The level of incongruence among individual gene trees and the two multigenic trees was first assessed by Shimodaira and Hasegawa tests [60]. The Shimodaira and Hasegawa test, based on sequence alignments, was used to compare majority-rule consensus tree topologies obtained with PAUP* for individual genes and the topologies of the supermatrix and BUCKy trees. Polytomies were randomly resolved by bipartitions using the *multi2di *function implemented in the APE package [61] of R 2.9.1 [62]. This was done because polytomies were strongly penalized in the log-likelihood score. Indeed, if polytomies are left unresolved it is not possible to determine whether significance of Shimodaira and Hasegawa tests was due to the fact that an alternative topology was more (or less) likely than a given tested topology or simply because it was more (respectively less) resolved. In all cases, when polytomies were kept the supermatrix and BUCKy trees resulted in higher log-likelihoods, consistent with the fact they are fully resolved. Shimodaira and Hasegawa tests were run using a GTR+Γ+I model in the BASEML program implemented in PAML 4.1 [63].

In addition, we used the χ^2 ^test of the PhySIC_IST preprocess [56] to identify triplets of leaves observed in the multigenic trees that were strongly rejected by the 27 bootstrap gene-tree collections. A strong rejection was defined as follows: denoting *R_s _*the set of triplets of a multigenic tree (supermatrix or BUCKy), and *R_b _*the set of triplets of the 2,700 bootstrap gene trees (100 per locus), a triplet of *R_s _*was said to be strongly rejected if it contradicted at least one triplet of *R_b _*and failed the χ^2 ^test described in [56] with a threshold of 0.9. Using this procedure we counted the number of strongly rejected triplets a taxon belongs to.

To quantify the degree of incongruence between individual gene trees and the two multigenic trees, we defined a triplet-based distance between a given multigenic tree (*T_s_*) and the forest (*F_j_*) of 100 bootstrap trees obtained for locus *j*. To put it simply, the triplet distance represented the percentage of triplets that were resolved differently by a multigenic tree (supermatrix or BUCKy) and a given gene tree. In order to separate the signal of this locus from potential stochastic errors, we focused on triplets that appeared more than 50% of times in *F_j_*. This threshold has the advantage of keeping one and only one resolution per group of 3 species. Defining a threshold at 60% does not qualitatively alter our results (results not shown).

We denoted  the number of retained triplets of *F_j _*that had the same resolution as *T_s_*, and  the number of retained triplets of *F_j _*with a different resolution. We defined the distance between the tree *T_s _*and the forest *F_j_*, denoted *d*(*T_s_, F_j_*), as the triplet fit dissimilarity (1 minus the triplet fit similarity [64]) between the triplet set of *T_s _*and the retained triplets of *F_j_*:(2)

Using similar procedures, we computed the triplet distance between all pairs of the 21 individual loci located on chromosome 3. We defined a triplet-based distance between each pair of forests *F_i _*and *F_j_*, where *F_i _*and *F_j _*were, respectively, the forests of 100 bootstrap trees obtained for loci *i *and *j*. As above, we focused on triplets that appeared more than 50% of times in each forest in order to eliminate potential stochastic errors. The distance *d*(*F_i_, F_j_*) between *F_i _*and *F_j _*is defined as:(3)

In this way, we obtained a symmetric distance matrix (*M*) with 21 rows and 21 columns, where each entry *M_ij _*contained the triplet distance between loci *i *and *j*. This matrix was used in the analysis of gene-tree incongruence and recombination (see below).

### Analyses of Patterns of Incongruence

In order to understand the origin of incongruences, we correlated triplet distances between individual loci and the multigenic trees (*d*(*T_s_, F_j_*) in equation 2) to relevant phylogenetic parameters, including alignment length, average evolutionary rate (estimated with the super-distance matrix method [65]), and shape parameter α of the gamma distribution (obtained in ML analyses of individual loci). We also tested if incongruences were positively correlated with recombination by using the 21 loci located on chromosome 3. This correlation is expected whatever the origin of tree incongruence. Indeed, following interspecific hybridization, recombination is necessary for genes of one species to introgress into the genome of the other species. Alternatively, because the effective population size is expected to be smaller in low than in high recombining regions [66,67], coalescence is expected to be quicker and lineage sorting more complete when recombination is low. We thus tested if the triplet distance was lower in centromeric than in telomeric regions by fitting a quadratic regression of *d*(*T_s_, F_j_*) on the genetic distance. We performed the same analyses on the aforementioned phylogenetic parameters because recombination could affect incongruences indirectly through these parameters (e.g., higher evolutionary rates in high recombining regions).

In addition, we tested whether the distribution of incongruences differed significantly between centromeric and telomeric loci located on chromosome 3. To this end, we estimated the triplet distance per pair of loci by distinguishing chromosome arms (short, long) and regions (centromere, telomere). Note that we did not mix loci located on different arms. Then, we obtained the difference in medians of the two distributions. To test whether this difference was statistically significant, we performed 10,000 replicates by permuting loci on each arm and recalculated the difference in medians at each permutation. The median difference observed with the actual dataset was compared with those observed in the permutated datasets.

Finally, closely linked loci more likely share a common genealogical history than distant loci [13]. To test this hypothesis we constructed a matrix of genetic distance between pairs of loci for the 21 genes located on chromosome 3. We correlated this matrix with the matrix of incongruences by pairs (*M_ij_*) and tested the significance of the correlation by performing 10,000 permutations of locus locations on each chromosome arm (avoiding permutation from one arm to another).

Statistical analyses were performed with R 2.9.1 [62] and analysis of the distribution of incongruences in centromeric and telomeric loci was performed with *Mathematica *[68].

## Results

### Numerous Incongruences among Individual Gene Trees

The best models describing the evolution of individual loci are presented in Table 3 and the corresponding trees in Additional files 2, 3, 4, 5, 6, 7, 8, 9, 10, 11, 12, 13, 14, 15, 16, 17, 18, 19, 20, 21, 22, 23, 24, 25, 26, 27 and 28, Figures S1-S27. Phylogenetic reconstructions using individual loci produce variable topologies. Often, relationships among genera and species are incongruent among individual loci. The positions of *Pseudoroegneria *and *Hordeum *are not stable among individual gene trees: in some cases, *Pseudoroegneria *branches in basal positions (e.g., LOC_Os01g01790, LOC_Os01g09300, LOC_Os01g24680, LOC_Os01g55530, LOC_Os01g56630, LOC_Os01g62900, *eIFiso4E*), whereas in other cases it branches within more recently diverging clades (e.g., LOC_Os01g11070, LOC_Os01g13200, LOC_Os01g39310, LOC_Os01g53720, LOC_Os01g61720, LOC_Os01g68770, LOC_Os01g73790, *CRTISO, PinA, PSY2, MATK*). Likewise, *Hordeum*, a genus thought to be one of the deepest among Triticeae [29,30,33], sometimes branches into quite terminal positions (LOC_Os01g01790, LOC_Os01g11070, LOC_Os01g21160, LOC_Os01g24680, LOC_Os01g37560, LOC_Os01g48720, LOC_Os01g53720, LOC_Os01g55530, LOC_Os01g56630, LOC_Os01g68770, LOC_Os01g70670, LOC_Os01g73790, *PSY2, eIFiso4E*, and *CRTISO*). Several other odd relationships involving different taxa are displayed by individual gene trees (Additional files 2, 3, 4, 5, 6, 7, 8, 9, 10, 11, 12, 13, 14, 15, 16, 17, 18, 19, 20, 21, 22, 23, 24, 25, 26, 27 and 28, Figures S1-S27). In general, individual gene trees have shorter internal branches than terminal branches (i.e., low treeness). In addition, support values (bootstrap values and posterior probabilities) of deeper nodes are weaker than those of more recent nodes. Similar observations were made in previous studies [22].

### Multigenic Analyses: a more Resolved Picture

**Figure 1:**
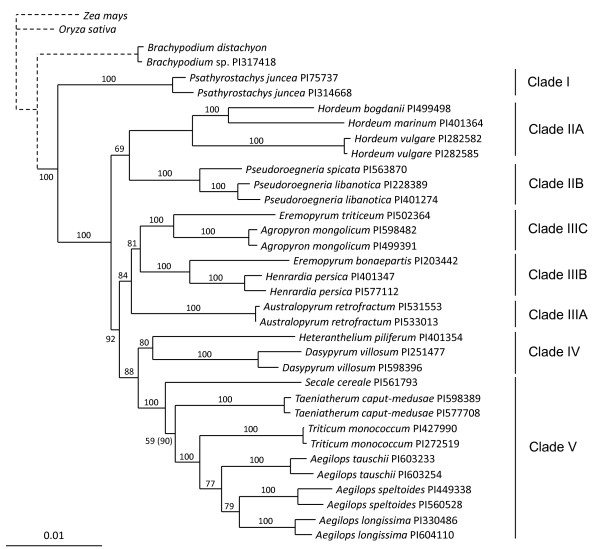
**Supermatrix phylogeny of Triticeae**. Phylogenetic tree inferred with the concatenation of 27 loci (~25 Kb). Bootstrap values are given in percentage. Maximal posterior probability (100%) for all nodes except one (indicated in brackets). Note that branch lengths of the outgroups are divided by 10 (dotted lines) in order to zoom in Triticeae.

The supermatrix tree obtained with the concatenation of all loci (~25 Kb) provides a much more resolved picture than individual gene trees. ML and Bayesian analyses are consistent and produce very similar trees. According to these trees, we distinguished 5 to 7 clades depending on posterior probability or bootstrap supporting values (Figure 1). The first divergent group within Triticeae is *Psathyrostachys *(clade I), followed by *Hordeum *(clade IIA) and *Pseudoroegneria *(clade IIB). The internal branches are quite short compared with the terminal branches, suggesting that cladogenesis occurred in rapid succession. Two well-supported clades diverge at this point. The first is formed by *Australopyrum *(clade IIIA), *Henrardia *and *Eremopyrum bonaepartis *(clade IIIB), and *Agropyrum *and *E. triticeum *(clade IIIC). The second consists of *Dasypyrum *and *Heteranthelium *(clade IV), on the one hand, and *Secale, Taeniatherum, Triticum *and *Aegilops *(clade V), on the other hand.

**Figure 2:**
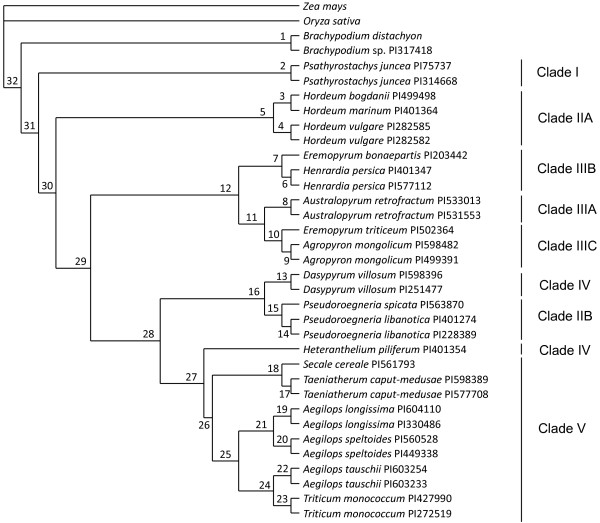
**Primary concordance tree of Triticeae**. Phylogenetic tree inferred with BUCKy. Splits are presented in branches. Concordance factors for splits are presented in Table 4. Clades named as in Figure 1.

BUCKy retrieves a unique topology irrespective of the different a priori levels of incongruence (α varying from 0.1 to 100), although mean sample-wide concordance factors (i.e., the proportion of the dataset that supports a bipartition) diminish with increasing α (Figure 2, Table 4). There is agreement between the estimated sample-wide and the extrapolated genome-wide concordance factors (i.e., the proportion of the whole genome that agrees with a given bipartition) and both concordance factors are higher in terminal than in deeper branches. The BCF tree is congruent with the supermatrix tree in several respects. First, *Psathyrostachys *(clade I) and then *Hordeum *(clade IIA) are the first divergent genera within Triticeae. Second, clade V is retrieved although branching within this clade changes relative to the supermatrix tree: *Secale *and *Taeniatherum *branch together, *T. monococcum *branches sister to *Ae. tauschii*, and *Ae. speltoides *and *Ae. longissima *group together. Third, monophyly of clade III is confirmed in this analysis although with alternative branching: *Henrardia *and *E. bonaepartis *(clade IIIB) are the first divergent taxa, and *Australopyrum *(clade IIIA) branches sister to *Agropyron *and *E. triticeum *(clade IIIC). However, a major discrepancy between this tree and the supermatrix tree is worth noting: *Pseudoroegneria *does not group with *Hordeum *but sister to *Dasypyrum*. Consequently, *Heteranthelium *branches at the base of clade V and these two new inferred clades (*Pseudoroegneria-Dasypyrum *and *Heteranthelium*-clade V) are closely related to each other. Despite differences among the supermatrix and BUCKy trees, the resolution and support gained with multigenic approaches compared with single-locus analyses are remarkable. Differences among trees are mainly due to uncertainty in the position of *Pseudoroegneria*.

**Table 4:** Primary concordance factors

Splits	Sample-wide CF	Genome-wide CF (α = 0.5)	Genome-wide CF (α = 10)
1	0.322 (0.259, 0.407)	0.321 (0.149, 0.522)	0.239 (0.109, 0.399)
2	0.814 (0.704, 0.889)	0.811 (0.616, 0.950)	0.598 (0.423, 0.760)
3	0.446 (0.333, 0.556)	0.445 (0.232, 0.664)	0.330 (0.168, 0.510)
4	0.486 (0.444, 0.556)	0.484 (0.294, 0.677)	0.359 (0.209, 0.523)
5	0.196 (0.111, 0.296)	0.195 (0.050, 0.390)	0.143 (0.036, 0.292)
6	0.780 (0.704, 0.815)	0.777 (0.588, 0.919)	0.573 (0.404, 0.732)
7	0.378 (0.259, 0.481)	0.377 (0.180, 0.592)	0.276 (0.128, 0.450)
8	0.562 (0.481, 0.630)	0.560 (0.364, 0.747)	0.414 (0.257, 0.581)
9	0.261 (0.185, 0.333)	0.260 (0.105, 0.452)	0.195 (0.077, 0.345)
10	0.128 (0.074, 0.185)	0.128 (0.023, 0.293)	0.094 (0.017, 0.218)
11	0.043 (0.000, 0.074)	0.042 (0.000, 0.173)	0.031 (0.000, 0.135)
12	0.046 (0.000, 0.074)	0.046 (0.000, 0.177)	0.034 (0.000, 0.136)
13	0.523 (0.444, 0.593)	0.521 (0.321, 0.715)	0.386 (0.228, 0.554)
14	0.543 (0.444, 0.630)	0.541 (0.332, 0.744)	0.401 (0.236, 0.576)
15	0.438 (0.333, 0.519)	0.437 (0.240, 0.641)	0.320 (0.170, 0.489)
16	0.071 (0.000, 0.111)	0.071 (0.000, 0.215)	0.052 (0.000, 0.162)
17	0.825 (0.741, 0.889)	0.822 (0.636, 0.954)	0.606 (0.435, 0.765)
18	0.059 (0.000, 0.148)	0.059 (0.000, 0.218)	0.043 (0.000, 0.164)
19	0.555 (0.481, 0.630)	0.553 (0.355, 0.742)	0.409 (0.251, 0.576)
20	0.562 (0.481, 0.630)	0.560 (0.356, 0.754)	0.414 (0.252, 0.586)
21	0.125 (0.037, 0.185)	0.125 (0.014, 0.294)	0.091 (0.010, 0.219)
22	0.568 (0.519, 0.593)	0.566 (0.369, 0.750)	0.418 (0.261, 0.584)
23	0.375 (0.296, 0.444)	0.374 (0.195, 0.570)	0.278 (0.141, 0.438)
24	0.056 (0.000, 0.111)	0.055 (0.000, 0.197)	0.041 (0.000, 0.151)
25	0.010 (0.000, 0.037)	0.010 (0.000, 0.094)	0.007 (0.000, 0.071)
26	0.031 (0.000, 0.037)	0.031 (0.000, 0.146)	0.023 (0.000, 0.116)
27	0.025 (0.000, 0.037)	0.025 (0.000, 0.135)	0.019 (0.000, 0.105)
28	0.028 (0.000, 0.037)	0.028 (0.000, 0.140)	0.020 (0.000, 0.110)
29	0.032 (0.000, 0.074)	0.032 (0.000, 0.157)	0.023 (0.000, 0.120)
30	0.067 (0.037, 0.111)	0.067 (0.003, 0.210)	0.049 (0.002, 0.159)
31	0.102 (0.037, 0.185)	0.102 (0.007, 0.272)	0.074 (0.005, 0.204)
32	0.199 (0.074, 0.333)	0.199 (0.038, 0.419)	0.150 (0.030, 0.318)

Mean concordance factors (CF) with 95% confidence intervals (between brackets) for the BUCKy tree. Splits are shown in Figure 2.

**Figure 3:**
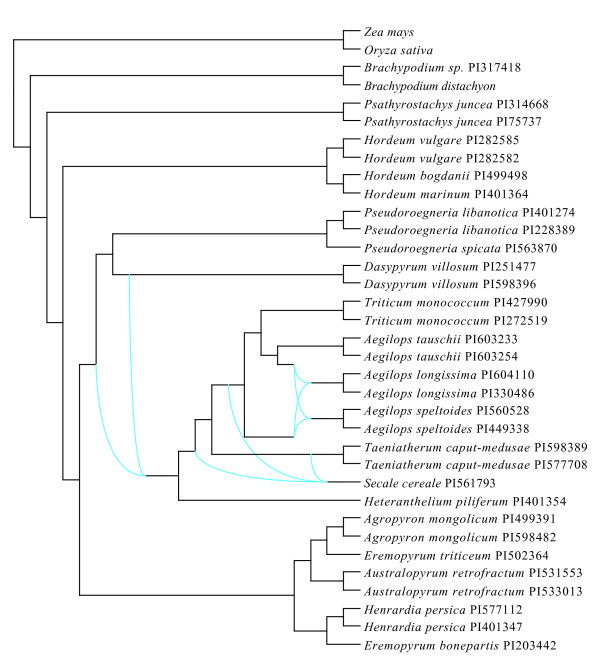
**Multigenic network of Triticeae**. Network obtained from the 27 individual gene trees modified with PhySIC_IST [56] using a correction threshold of 0.9 (see details in Methods).

The multigenic network displays most of the relationships present in the supermatrix and BUCKy trees (Figure 3). In addition, it points to the less resolved parts of the phylogeny that mainly correspond to nodes with low support. *Psathyrostachys *and *Hordeum *are the deepest genera of Triticeae. Their divergence occurs in a tree-like manner as do relationships within clade III. Note that the topology of clade III is the same between the multigenic network and the BUCKy tree. Uncertainties for inter-clade relationships mainly involve *Dasypyrum *and *Heteranthelium *but only few alternatives are proposed by the network analysis. Finally, branching of most species in clade V is quite variable and this instability is better taken into account by the network than by any of the multigenic trees (supermatrix or BUCKy). Overall, the network analysis reveals a general tree-like divergence history of the Triticeae with local episodes of reticulate evolution.

### Patterns of Incongruence among Trees

One of the most puzzling results obtained in this study is the numerous incongruences among individual gene trees. In most cases, Shimodaira and Hasegawa tests confirm that, regarding a given locus alignment, the corresponding gene tree has a significantly higher log-likelihood than that of other individual gene trees. However, in most cases, differences between individual gene trees and the two multigenic trees (supermatrix and BUCKy) are not statistically significant (Table 5). This suggests that the splitting histories of species lineages depicted by the supermatrix or BUCKy trees are reasonable compromises of individual gene tree scenarios.

**Table 5:** Shimodaira and Hasegawa tests among individual gene trees and the two multigenic trees (supermatrix and BUCKy)

	1	2	3	4	5	6	7	8	9	10	11	12	13	14	15	16	17	18	19	20	21	22	23	24	25	26	27	28
1	ns	*	*	*	*	*	*	*	*	*	*	*	*	*	*	*	*	*	*	*	*	*	*	*	*	*	*	*
2	*	ns	ns	*	*	*	*	*	*	ns	*	*	*	*	*	ns	*	*	*	*	*	*	*	*	*	*	*	*
3	*	*	ns	*	*	*	*	*	*	*	*	*	*	*	*	*	*	*	*	*	*	*	*	*	*	*	*	*
4	*	*	*	ns	*	*	*	*	*	*	*	*	*	*	*	*	*	*	*	*	*	*	*	*	*	*	*	*
5	*	*	ns	*	ns	*	*	*	*	*	*	*	*	*	*	*	*	*	*	*	*	*	*	*	*	*	*	*
6	*	ns	ns	*	*	ns	*	*	*	*	*	*	*	*	*	*	*	*	*	*	*	*	*	*	*	*	*	*
7	*	*	*	*	*	*	ns	*	*	ns	ns	*	*	*	*	*	*	*	ns	*	*	*	ns	*	*	ns	*	*
8	*	*	*	*	*	*	*	ns	*	*	*	*	*	*	*	*	*	*	*	*	*	*	*	*	*	*	*	*
9	*	*	*	*	*	*	*	*	ns	*	*	*	*	*	*	*	*	*	*	*	*	*	*	*	*	*	*	*
10	*	*	*	*	*	*	*	*	*	ns	*	*	*	*	*	*	*	*	*	*	*	*	*	*	*	*	*	*
11	*	*	*	*	*	*	*	*	*	ns	ns	*	*	*	*	ns	*	*	*	*	*	*	*	*	*	*	*	*
12	*	ns	ns	*	*	*	ns	*	*	ns	*	ns	*	*	*	*	*	*	*	*	*	*	*	*	*	*	*	*
13	*	*	ns	*	*	*	*	*	*	*	*	*	ns	*	*	*	*	*	*	*	*	*	*	*	*	*	ns	*
14	*	ns	ns	*	*	ns	ns	*	*	*	*	*	*	ns	*	*	ns	*	ns	*	*	*	*	*	ns	*	ns	*
15	*	ns	ns	*	*	ns	ns	*	*	*	ns	*	*	*	ns	*	ns	*	*	*	ns	*	ns	*	ns	*	ns	*
16	*	*	*	*	*	*	*	*	*	*	*	*	*	*	*	ns	*	*	*	*	*	*	*	*	*	*	*	*
17	*	*	*	*	*	*	*	*	*	*	*	*	*	*	*	ns	*	*	*	*	*	*	*	*	*	*	*	*
18	*	ns	ns	*	*	ns	*	ns	*	*	*	*	*	*	*	*	*	ns	*	*	*	*	ns	*	ns	*	*	*
19	*	*	*	*	*	*	*	*	*	*	*	*	*	*	*	ns	*	*	ns	*	*	*	*	*	*	*	*	*
20	*	*	*	*	*	*	*	*	*	ns	*	*	*	*	*	*	*	*	*	ns	*	*	*	*	*	*	*	*
21	*	*	*	*	*	*	*	*	*	*	*	*	*	*	*	*	*	*	*	*	ns	*	*	*	*	*	*	*
22	*	*	*	*	*	*	*	*	*	ns	*	*	*	*	*	*	*	*	*	*	*	ns	*	*	*	*	*	*
23	*	*	*	*	*	*	*	*	*	*	*	*	*	*	*	*	*	*	*	*	*	*	ns	*	*	*	*	*
24	*	ns	ns	*	*	*	ns	ns	*	ns	ns	*	ns	*	*	ns	*	*	ns	ns	ns	*	ns	ns	ns	*	ns	*
25	*	*	*	*	*	*	*	*	*	*	*	*	*	*	*	*	*	*	*	*	*	*	*	*	ns	*	*	*
26	*	*	*	*	*	*	*	*	*	*	*	*	*	*	*	*	*	*	*	*	*	*	*	*	*	ns	*	*
27	*	*	*	*	*	*	*	*	*	*	*	*	*	*	*	*	*	*	*	*	*	*	*	*	*	*	ns	*
29	*	ns	ns	*	ns	ns	ns	ns	ns	ns	ns	ns	ns	ns	ns	ns	ns	ns	ns	ns	ns	ns	ns	ns	ns	ns	ns	ns
30	ns	ns	ns	*	ns	ns	ns	ns	*	ns	ns	ns	ns	ns	ns	ns	ns	ns	ns	ns	ns	*	ns	ns	ns	ns	ns	ns

Alignments are presented in columns and tree topologies in rows. *: *P *< 0.05; ns: *P *> 0.05.

(1) CRTISO; (2) eifiso4E; (3) LOC_Os01g01790; (4) LOC_Os01g09300; (5) LOC_Os01g11070; (6) LOC_Os01g13200; (7) LOC_Os01g19470; (8) LOC_Os01g21160; (9) LOC_Os01g24680; (10) LOC_Os01g37560; (11) LOC_Os01g39310; (12) LOC_Os01g48720; (13) LOC_Os01g53720; (14) LOC_Os01g55530; (15) LOC_Os01g56630; (16) LOC_Os01g60230; (17) LOC_Os01g61720; (18) LOC_Os01g62900; (19) LOC_Os01g67220; (20) LOC_Os01g68770; (21) LOC_Os01g70670; (22) LOC_Os01g72220; (23) LOC_Os01g73790; (24) MATK; (25) PinA; (26) PinB; (27) PSY2; (28) all loci; (29) supermatrix topology; (30) BUCKy topology.

In order to quantify topological incongruences, we estimated triplet distances among individual gene trees, as well as between each individual gene tree and the two multigenic trees, as described in Incongruence Quantification in the Methods section. The average triplet distance between individual gene trees (in absolute value) is 0.53 ± 0.14 (mean ± SD; range: 0.10-0.88). The average triplet distance between individual gene trees and the supermatrix tree is 0.21 ± 0.10 (0.08-0.50) and the average triplet distance between individual gene trees and the BUCKy tree is 0.25 ± 0.09 (0.07-0.43). In addition, we counted, for each accession, the number of triplets that are observed in individual gene trees and strongly rejected by the two multigenic trees. Excepting the two *Psathyrostachys *accessions, all other taxa are involved in several strongly rejected triplets (Table 6). *Pseudoroegneria *is often the most incongruent genus (13% or 24% of all incongruences according to the BUCKy or supermatrix tree, respectively). Whereas *Aegilops*/*Triticum *species are not especially incongruent with the supermatrix tree (16%), they are highly incongruent with the BUCKy tree (35%). Conversely, *Hordeum *is involved in many incongruent triplets when the supermatrix tree is used (20%) but is only involved in as few as 1% of incongruent triplets when the BUCKy tree is considered. Results are similar when we remove the outgroups (*Zea, Oryza *and *Brachypodium*) and re-root the trees with *Psathyrostachys *(results not shown). This demonstrates that incongruences are not due to the difficult positioning of the root of the trees.

**Table 6:** Number of incongruent strongly rejected triplets per accession

Species	Accession	Supermatrix	BUCKy
*Pseudoroegneria libanotica*	PI 228389	109	110
*Pseudoroegneria libanotica*	PI 401274	73	111
*Pseudoroegneria spicata*	PI 563870	68	143
*Hordeum bogdanii*	PI 499498	62	19
*Hordeum vulgare*	PI 282582	58	5
*Hordeum vulgare*	PI 282585	52	0
*Australopyrum retrofractum*	PI 531553	45	78
*Australopyrum retrofractum*	PI 533013	39	82
*Eremopyrum bonaepartis*	PI 203442	39	101
*Hordeum marinum*	PI 401364	39	12
*Agropyron mongolicum*	PI 598482	36	99
*Secale cereale*	PI 561793	36	111
*Triticum monococcum*	PI 272519	34	137
*Dasypyrum villosum*	PI 598396	26	95
*Dasypyrum villosum*	PI 251477	25	103
*Taeniatherum caput-medusae*	PI 577708	25	89
*Aegilops speltoides*	PI 560528	24	82
*Eremopyrum triticeum*	PI 502364	24	97
*Henrardia persica*	PI 401347	22	141
*Taeniatherum caput-medusae*	PI 598389	22	89
*Triticum monococcum*	PI 427990	21	157
*Aegilops longissima*	PI 604110	20	105
*Heteranthelium piliferum*	PI 401354	20	97
*Aegilops tauschii*	PI 603233	19	156
*Aegilops speltoides*	PI 449338	18	96
*Agropyron mongolicum*	PI 499391	18	85
*Henrardia persica*	PI 577112	18	141
*Aegilops longissima*	PI 330486	17	97
*Aegilops tauschii*	PI 603254	17	152
*Psathyrostachys juncea*	PI 314668	0	0
*Psathyrostachys juncea*	PI 75737	0	0

Incongruent triplets calculated between individual loci and the two multigenic trees (supermatrix and BUCKy). Rows are decreasingly sorted according to the supermatrix tree.

### The Effect of Recombination on Incongruences

**Figure 4:**
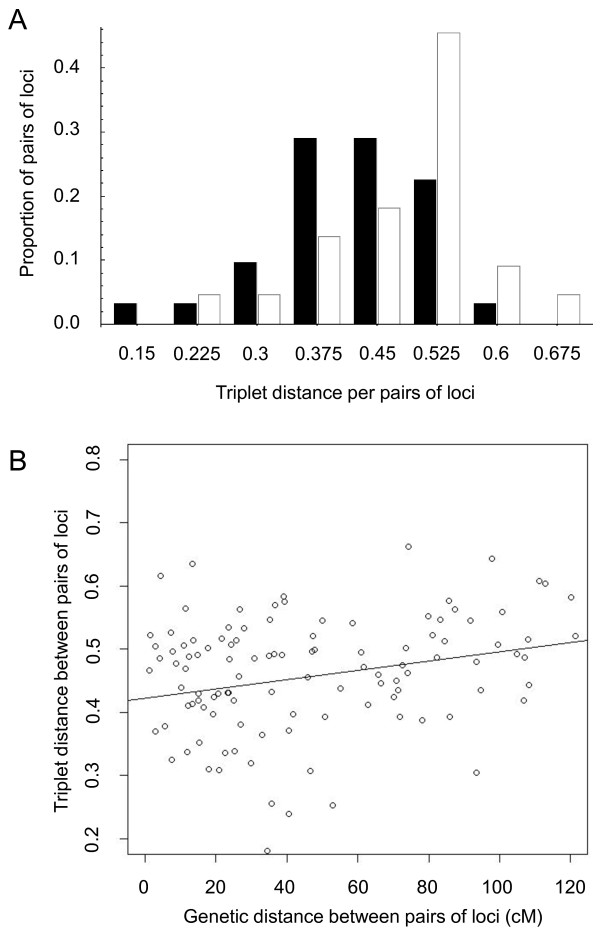
**Effect of recombination on incongruences**. A. Incongruence level of loci located in centromeres (filled bars) and telomeres (open bars). B. Correlation between the triplet distance and genetic distance between pairs of loci. Only loci located on chromosome 3 are depicted.

**Figure 5:**
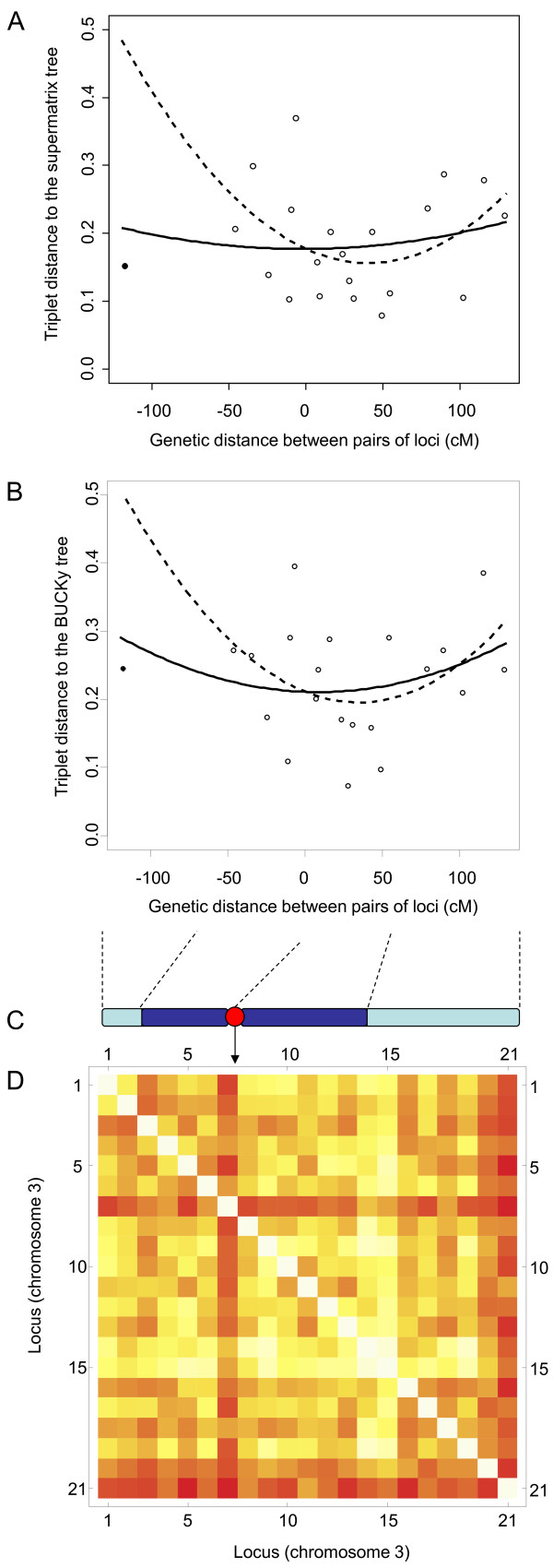
**Effect of recombination on incongruences**. Relationship between the triplet distance between individual gene trees and the two multigenic trees (supermatrix tree in A; BUCKy tree in B) as a function of the genetic distance between genes located on chromosome 3. The triplet distance between individual gene trees and the multigenic trees is the percentage of triplets of accessions that were resolved differently by a multigenic tree and a given gene tree. Solid line: best fit using all points; dashed line: best fit without a potential outlier (filled point). The genetic distance is connected to the chromosomal position according to the schematic diagram presented in C (red point: centromere; dark blue: centromeric regions; light blue: telomeric regions). D. Degree of incongruence among pairs of loci relative to the genetic distance on chromosome 3. Colors represent the degree of incongruence (white: no incongruence; red: strongest incongruence).

We performed several tests to understand the origin of incongruences among gene trees. First, we tested if variation in incongruence could be explained by the nature of the phylogenetic signal. After correlating the triplet distance between individual genes and the two multigenic trees (supermatrix and BUCKy) with relevant phylogenetic parameters per locus, we only detect a significant positive correlation between the average evolutionary rate and triplet distance separating individual gene trees from the supermatrix tree (Spearman's rho = 0.42, *P *= 0.03), although not with the BUCKy tree (rho = 0.21, *P *= 0.28). Next, we investigated the effect of recombination. Recombination does not significantly affect any phylogenetic parameter (*P *> 0.5 in all correlations; results not shown). In contrast, it affects incongruences in three ways. First, incongruences are significantly greater in telomeric than in centromeric loci (respective medians = 0.511 and 0.429, *P *= 0.028; Figure 4A). Second, loci located close together on the chromosome tend to have more similar genealogical histories than distant loci (Figure 4B; rho = 0.22, *P *= 0.026). Third, although the statistical significance is low (possibly because of the small number of genes we sampled), incongruences between individual gene trees and the two multigenic trees tend to increase with the genetic distance between pairs of loci, that is, with the likelihood of recombination (*P *= 0.09 for the supermatrix tree; *P *= 0.10 for the BUCKy tree; in the two cases we removed one potential outlier; Figure 5). Note that similar qualitative patterns are observed when using a more restrictive cut-off to keep incongruences (triplets that appeared more than 60% of times in the 100 bootstrap trees rather than 50% of times), although the statistical significance disappeared (results not shown). Again, this is possibly due to the limited number of genes we sampled.

## Discussion

### Multigenic Phylogeny of Triticeae

Up to date, morphological and molecular analyses have failed to infer a reliable phylogeny of the Triticeae. Many previous phylogenetic reconstructions were based on a limited number of genes, in most cases only one (see references above). The many conflicts among published trees, combined with poor resolution of branching among genera and species, prevented a clear picture of the relationships among members of this tribe from emerging. Moreover, it was not possible to conclude whether reticulate evolution is the dominant rule in this tribe, so that reconstructing a resolved phylogeny is hopeless, or whether multigenic approaches could solve the problem. Thanks to the largest dataset used so far in this tribe (27 genes), we show that combining information from several loci located on different chromosomes and cellular compartments (nucleus and chloroplast) enable the identification of major clades. Although the branching position of some groups remains uncertain (e.g., *Pseudoroegneria*), the two multigenic trees and the multigenic network enabled us to resolve most parts of the Triticeae phylogeny.

Some incongruence persists in our analyses. They are represented in the phylogeny by low support values (bootstrap, posterior support and concordance factors) and are summarized within the multigenic network. Figures 1, 2 and 3 show that *Psathyrostachys *branches sister to the remaining Triticeae, followed by the sequential branching of *Hordeum*. Some previously published phylogenies recognized the early divergence of *Psathyrostachys *and *Hordeum*, including nuclear [23,27,33] and chloroplastic DNA analyses [29,30], although many other studies disagreed [24,26,28,32,33]. Here, *Psathyrostachys *is involved in no incongruence (Table 6) and the branch leading to the rest of the tribe is among the longest internal branches. This clearly indicates that this genus is the sister group of all other Triticeae.

Our dataset does not allow us to resolve the position of *Pseudoroegneria*. No study has raised the possibility that it branches out with *Hordeum*, as proposed by the supermatrix tree, and only one study suggested that *Pseudoroegneria *could group with *Dasypyrum*, as proposed by the BUCKy tree, although support for this relationship was low [69]. Other studies proposed that *Pseudoroegneria *could branch sister to *Taeniatherum *and/or *Australopyrum *[23,24,33], *Heteranthelium *[26] or *Aegilops *[32]. In other cases its branching pattern was unstable [22,29] and it was even considered paraphyletic [30,70]. Consistent with the difficult positioning of this genus, the supermatrix tree groups it with *Hordeum *with a rather weak bootstrap support (0.69), although maximal posterior probability (1.00), conflicting with the BUCKy tree. More strikingly, the three *Pseudoroegneria *accessions are, in general, involved in more incongruent triplets than other accessions (Table 6). This could be due to a strong capacity of introgression during divergence of this group and/or a large ancestral population size. In agreement with the first hypothesis, *Pseudoroegneria *and *Hordeum *hybridized and were at the origin of the species-rich allotetraploid genus *Elymus *[23,30]. If recent interspecific introgression were responsible for the incongruence pattern we observed it would likely proceed via polyploids because all genera investigated here are currently intersterile [71-74]. Polyploids could serve as bridges of genes between diploid species [22]. Alternatively, if rapid radiation followed by incomplete lineage sorting were the main factors contributing to incongruence, one would expect deep branches to be short and less supported than external branches. This is basically what we observe in individual gene trees and the supermatrix tree. The relatively strong support obtained in the supermatrix analysis is due to accumulation of phylogenetic signals when concatenating all genes. Hence, it could be that both factors, hybridization and incomplete lineage sorting of ancestral polymorphism, contributed to the pattern of incongruence and the unstable positioning of *Pseudoroegneria*.

Phylogenetic positions of all other genera and species varied in previous studies and no consensus emerged. In the present study we find strong support for clades III and V, although branching order varied depending on the multigenic tree. Overall, our results suggest rapid radiation following -or concomitant with- divergence of clade III (grouping *Agropyron, Australopyrum, Eremopyrum *and *Henrardia*).

### Incongruence and Recombination

Our results provide strong evidence of incongruence among individual gene trees, unraveling a complex biological reality where different portions of the genome exhibit different histories (their own evolutionary histories). We analyze the pattern of incongruence and pinpoint the role of recombination and gene location on it. We demonstrate that physically close loci are more likely to share a common history than distant loci. More interestingly, loci located in centromeric regions tend to be more congruent with one another than loci located in telomeric regions. A similar correlation was found in *Drosophila *at the kilobase scale, the scale of linkage disequilibrium in this group [13]. In contrast, the mosaics of conflicting genealogies observed in *Oryza *(rice) were randomly distributed across the genome [75]. It could be surprising that the correlation we observe holds at the scale of the whole chromosome 3 (~1 Gb; [38]). Several non-exclusive reasons could explain this pattern. First, the recombination gradient along chromosomes is very steep in all Triticeae, including wheat [42,43,45], rye [76] and *Aegilops speltoides *[44]. For instance, along chromosome 3B in bread wheat (*Triticum aestivum*), the cM/Mb ratio spans about two orders of magnitude (from 0.01 to 0.85; [41]). Accordingly, in bread and durum wheat (*T. aestivum *and *T. durum*, respectively), linkage disequilibrium decays slowly over several cM [77]. Despite the impressive chromosome size, linkage disequilibrium could be high in centromeric regions because recombination is strongly reduced (see [78] for a study on chromosome 3B). However, the level of linkage disequilibrium is low in barley [79]. This discrepancy highlights the need for studying linkage disequilibrium patterns in Triticeae in more detail. Second, centromeric genes may have a lower local effective size than telomeric genes because of hitchhiking effects due to the lack of recombination [66,67]. In agreement with this prediction, the levels of diversity positively correlate with the proxy of recombination in *Aegilops*: the RFLP polymorphism is 1.5 to 25 times higher in telomeric than in centromeric regions [80]. Consequently, ancient polymorphisms would be less completely sorted in genes located in high recombining than in low recombining regions. Finally, recombination could play an important role in introgressive events between species. For instance, genes located in high recombining regions would introgress easier than genes located in regions of low recombination.

There is no straightforward way to distinguish whether the overall pattern of incongruence in Triticeae is produced by incomplete lineage sorting or a form of introgression (e.g., gene flow proceeding via polyploids). Methods that enable introgression and incomplete lineage sorting to be distinguished [81] require the estimation of population sizes and divergence times for all branches of the species phylogeny. This information cannot be obtained in Triticeae without making strong assumptions. More knowledge about population parameters and divergence times is necessary to distinguish between these two sources of tree incongruence in this tribe.

## Conclusions

Our study contributes two important aspects for research in Triticeae in particular, and for the broad phylogenetic community in general. First, we show that in spite of strong tree conflicts not all clades of Triticeae are affected by introgression and/or incomplete lineage sorting. Notably, *Psathyrostachys, Hordeum *and genera in the clade III (including *Agropyron, Australopyrum, Eremopyrum *and *Henrardia*) diverge in a tree-like manner, a result that was not supported by previous studies. Because the evolution of *Pseudoroegneria *and genera in clades IV (*Dasypyrum *and *Heteranthelium*) and V (*Secale, Taeniatherum, Triticum *and *Aegilops*) is more reticulated than in other clades, the multigenic network better reflects their phylogenetic history than do the supermatrix or BUCKy trees. Second, we demonstrate that recombination could be an important evolutionary force in exacerbating the level of incongruence among gene trees. It would be worthwhile estimating the frequency of recombination of genes used in future phylogenetic studies in order to assess the generality of the pattern previously observed in *Drosophila *and now evidenced in Triticeae.

## Authors' contributions

JSE performed analyses, discussed results and wrote the paper. CS performed analyses, discussed results and wrote the paper. AC designed experiments and obtained sequence data. CG obtained sequence data. SS designed experiments and obtained sequence data. EJPD discussed results and wrote the paper. VR performed analyses, discussed results and wrote the paper. SG coordinated the project, performed analyses, discussed results and wrote the paper. JD coordinated the project and discussed results. All authors read and approved the final version of the paper.

## Supplementary Material

Additional file 1 **Primers used in the PCR amplification of each locus**. Table S1 with the sequence of each primer used during PCR amplification. Click here for file

Additional file 2 **Phylogenetic tree inferred with LOC_Os01g01790 sequences**. Figure S1 showing the phylogenetic tree inferred with locus LOC_Os01g01790. Click here for file

Additional file 3 **Phylogenetic tree inferred with LOC_Os01g09300sequences**. Figure S2 showing the phylogenetic tree inferred with locus LOC_Os01g09300. Click here for file

Additional file 4 **Phylogenetic tree inferred with LOC_Os01g11070 sequences**. Figure S3 showing the phylogenetic tree inferred with locus LOC_Os01g11070. Click here for file

Additional file 5 **Phylogenetic tree inferred with LOC_Os01g13200 sequences**. Figure S4 showing the phylogenetic tree inferred with locus LOC_Os01g13200. Click here for file

Additional file 6 **Phylogenetic tree inferred with LOC_Os01g19470 sequences**. Figure S5 showing the phylogenetic tree inferred with locus LOC_Os01g19470. Click here for file

Additional file 7 **Phylogenetic tree inferred with LOC_Os01g21160 sequences**. Figure S6 showing the phylogenetic tree inferred with locus LOC_Os01g21160. Click here for file

Additional file 8 **Phylogenetic tree inferred with LOC_Os01g24680 sequences**. Figure S7 showing the phylogenetic tree inferred with locus LOC_Os01g24680. Click here for file

Additional file 9 **Phylogenetic tree inferred with LOC_Os01g37560 sequences**. Figure S8 showing the phylogenetic tree inferred with locus LOC_Os01g37560. Click here for file

Additional file 10 **Phylogenetic tree inferred with LOC_Os01g39310 sequences**. Figure S9 showing the phylogenetic tree inferred with locus LOC_Os01g39310. Click here for file

Additional file 11 **Phylogenetic tree inferred with LOC_Os01g48720 sequences**. Figure S10 showing the phylogenetic tree inferred with locus LOC_Os01g48720. Click here for file

Additional file 12 **Phylogenetic tree inferred with LOC_Os01g53720 sequences**. Figure S11 showing the phylogenetic tree inferred with locus LOC_Os01g53720. Click here for file

Additional file 13 **Phylogenetic tree inferred with LOC_Os01g55530 sequences**. Figure S12 showing the phylogenetic tree inferred with locus LOC_Os01g55530. Click here for file

Additional file 14 **Phylogenetic tree inferred with LOC_Os01g56630 sequences**. Figure S13 showing the phylogenetic tree inferred with locus LOC_Os01g56630. Click here for file

Additional file 15 **Phylogenetic tree inferred with LOC_Os01g60230 sequences**. Figure S14 showing the phylogenetic tree inferred with locus LOC_Os01g60230. Click here for file

Additional file 16 **Phylogenetic tree inferred with LOC_Os01g61720 sequences**. Figure S15 showing the phylogenetic tree inferred with locus LOC_Os01g61720. Click here for file

Additional file 17 **Phylogenetic tree inferred with LOC_Os01g62900 sequences**. Figure S16 showing the phylogenetic tree inferred with locus LOC_Os01g62900. Click here for file

Additional file 18 **Phylogenetic tree inferred with LOC_Os01g67220 sequences**. Figure S17 showing the phylogenetic tree inferred with locus LOC_Os01g67220. Click here for file

Additional file 19 **Phylogenetic tree inferred with LOC_Os01g68770 sequences**. Figure S18 showing the phylogenetic tree inferred with locus LOC_Os01g68770. Click here for file

Additional file 20 **Phylogenetic tree inferred with LOC_Os01g70670 sequences**. Figure S19 showing the phylogenetic tree inferred with locus LOC_Os01g70670. Click here for file

Additional file 21 **Phylogenetic tree inferred with LOC_Os01g72220 sequences**. Figure S20 showing the phylogenetic tree inferred with locus LOC_Os01g72220. Click here for file

Additional file 22 **Phylogenetic tree inferred with LOC_Os01g73790 sequences**. Figure S21 showing the phylogenetic tree inferred with locus LOC_Os01g73790. Click here for file

Additional file 23 **Phylogenetic tree inferred with *eIFiso4E *sequences**. Figure S22 showing the phylogenetic tree inferred with locus *eIFiso4E*. Click here for file

Additional file 24 **Phylogenetic tree inferred with *CRTISO *sequences**. Figure S23 showing the phylogenetic tree inferred with locus *CRTISO*. Click here for file

Additional file 25 **Phylogenetic tree inferred with *PinA *sequences**. Figure S24 showing the phylogenetic tree inferred with locus *PinA*. Click here for file

Additional file 26 **Phylogenetic tree inferred with *PinB *sequences**. Figure S25 showing the phylogenetic tree inferred with locus *PinB*. Click here for file

Additional file 27 **Phylogenetic tree inferred with *PSY2 *sequences**. Figure S26 showing the phylogenetic tree inferred with locus *PSY2*. Click here for file

Additional file 28 **Phylogenetic tree inferred with *MATK *sequences**. Figure S27 showing the phylogenetic tree inferred with locus *MATK*. Click here for file
