# Supplementary material for: Multigenic phylogeny and analysis of tree incongruences in Triticeae (Poaceae)
Source: BMC Evol Biol. 2011 Jun 24;11:181. doi: 10.1186/1471-2148-11-181 (PMC3142523; doi:10.1186/1471-2148-11-181)
Supplement: Additional file 1 — Primers used in the PCR amplification of each locus. Table S1 with the sequence of each primer used during PCR amplification. [file 1471-2148-11-181-S1.PDF]

Table S1. Primers used in the PCR amplification of each locus. F: forward; R: reverse.

Locus	Primer	Primer sequence (5' to 3')	Position
LOC_Os01g01790	Os01790-UF	TCATAAACGTGGAGGTGCCCGAGGA	Exon 6
	Os01790-UR	GAACCAGGAAGCTCTGCTCAGGCAC	Exon 11
LOC_Os01g09300	Os09300-UF	TGGAGAGCTCGACATCGAGGACGAG	Exon 1
	Os09300-UR	TGCGTGTCCTTGTAGCGCTG	Exon 5
LOC_Os01g11070	Os11070-UF	TGCTATGCTTAACCAGTGGCTGC	Exon 3
	Os11070-UR	ACGATCGGTATAGTGCCGGCGAAAG	Exon 10
LOC_Os01g13200	Os13200-UF	CTACTTCGCCGTATCATCCGCGTCG	Exon 1
	Os13200-UR	AGTTCCTCATCTCTTGCTCGGTAGC	Exon 9
LOC_Os01g19470	Os19470-UF	TCTCAGTGTCTGCAAGCGGATGCCA	Exon 21
	Os19470-UR	TGGGTATGTGATGGAAGGCCAGACC	Exon 25
LOC_Os01g21160	Os21160-UF	GCAGCTCTGTGAGGTACAGAGCGAC	Exon 3
	Os21160-UR	TGTGCAGCAAGAGCAGCTCC	Exon 8
LOC_Os01g24680	Os24680-UF	AGAGGCACACCAACTGGGTC	Exon 8
	Os24680-UR	ACTGCATACCAGTAGCCACAGC	Exon 14
LOC_Os01g37560	Os37560-UF	GGATGGCAACAAGGACGACGCTGTC	Monoexonic
	Os37560-UR	TGTCTGGTAGGCTAGCCCCCATTGG	
LOC_Os01g39310	Os39310-UF	AGGCTCATGTGGTGTCAGTTCGTGC	Exon 5
	Os39310-UR	AGGAACCTGCTGATGTCACGTTGC	Exon 12
LOC_Os01g48720	Os48720-UF	TCGACAATGTCCTGCATCGGCTG	Exon 3
	Os48720-UR	TGTTCCTGCTGCGGAAGCGA	Exon 13
LOC_Os01g53720	Os53720-UF	CGTCGAGGATGATTTGCCCGACGA	Exon 1
	Os53720-UR	AGCTCAAGCATATCAGGCTGTGC	Exon 11
LOC_Os01g55530	Os55530-UF	AACAACACGCCTTCCACGGGTG	Monoexonic
	Os55530-UR	ACTGAGGGTTTCTCGAGCCGAGACC	
LOC_Os01g56630	Os56630-UF	AGCGCTGAAGGTGCGACAAGTC	Exon 6
	Os56630-UR	ACGAGGATGGTTCTTGAACGGCTG	Exon 14
LOC_Os01g60230	Os60230-UF	GCTCTCCATGCTCAAGTACGCCGTG	Exon 1
	Os60230-UR	ACCCTCTGAGGTTTGCAACGGCAC	Exon 10
LOC_Os01g61720	Os61720-UF	GTCTTCAGCTCCTCTGATCCGGAGG	Exon 1
	Os61720-UR	GGTTGATGGTGATTGCCGGCTTGG	Exon 5
LOC_Os01g62900	Os63690-UF	TACGAGCAGTACCTGCGGCT	Monoexonic
	Os63690-UR	TGCTGAACCCAGTAGTCGGCCAAG	
LOC_Os01g67220	Os67220-UF	ACATTGAGCTCATGGCCAAGTTGG	Exon 4
	Os67220-UR	TCGCCGTTCAAGAAGCGCGAGAAC	Exon 13
LOC_Os01g68770	Os68770-UF	ATTACCTGGCCACGGTGGACGT	Exon 2
	Os68770-UR	AGGTCAGGGCCATAGAACTGCTCG	Exon 7
LOC_Os01g70670	Os70670-UF	ACTGCACCTCTTTGGGGTGGTAGG	Exon 7
	Os70670-UR	ACAATTCAGCTTTGCGTCTGTGG	Exon 11
LOC_Os01g72220	Os72220-UF	TCATGCTCCGATGACACAACCGTG	Exon 4
	Os72220-UR	TGCATGCCAGAAGCACCTGAG	Exon 13
LOC_Os01g73790	Os73790-UF	TCTGCGAGAAGCTCATGGCCGAGAC	Exon 1

	Os73790-UR	GGCCAGACACATCTGCGTCTTGACG	Exon 9
eIFiso4E	eIFiso4E-F4	TCATCCTCCCCATCCCAAAACC	Exon 1
	eIFiso4E-F5	GACATGGCAGAGGTCGAAGC	Exon 1
	eIFiso4E-F6	AGTGGACCTTCTGGTACGACATCCA	Exon 1
	eIFiso4E-R1	ATCTCGGTAAAACACTACACGGC	Exon 5
	eIFiso4E-R5	TGTTGTAATCAAGGGATATTGCGAGG	Exon 5
CRTISO	CRTISO-UF	AGTCATACCAGACCCTTCTACG	Exon 2
	CRTISO-UR	GTGTCCCATCTTGTGGCATTTG	Exon 7
PinA	PinA-UF	TGCACACAGAAATCGTGCCACC	Monoexonic
	PinA-UR	CATACTGAAATGTTTGCTATAGACACCTCG	
PinB	PinB-UF	CATTTCTGTTGGCTCGCAAAGT	Monoexonic
	PinB-UR	GAGCACACTTATTTGTCACATGTTGTGG	
PSY2	PSY2-UF	ACTCCTGAAAGGCGCAAAG	Exon 3
	PSY2-UR	CCTCCTCCATTTATCCGTCA	Exon 5
MATK	MATK-UF	AACCCGGAACTAGTCGGATG	Monoexonic
	MATK-UR	CTCAATGGTAGAGTACTCGG	
