# Supplementary material for: Multigenic phylogeny and analysis of tree incongruences in Triticeae (Poaceae)
Source: BMC Evol Biol. 2011 Jun 24;11:181. doi: 10.1186/1471-2148-11-181 (PMC3142523; doi:10.1186/1471-2148-11-181)

Figure S2. Phylogenetic tree inferred with LOC_Os01g09300 sequences. Values in nodes are bootstrap values.

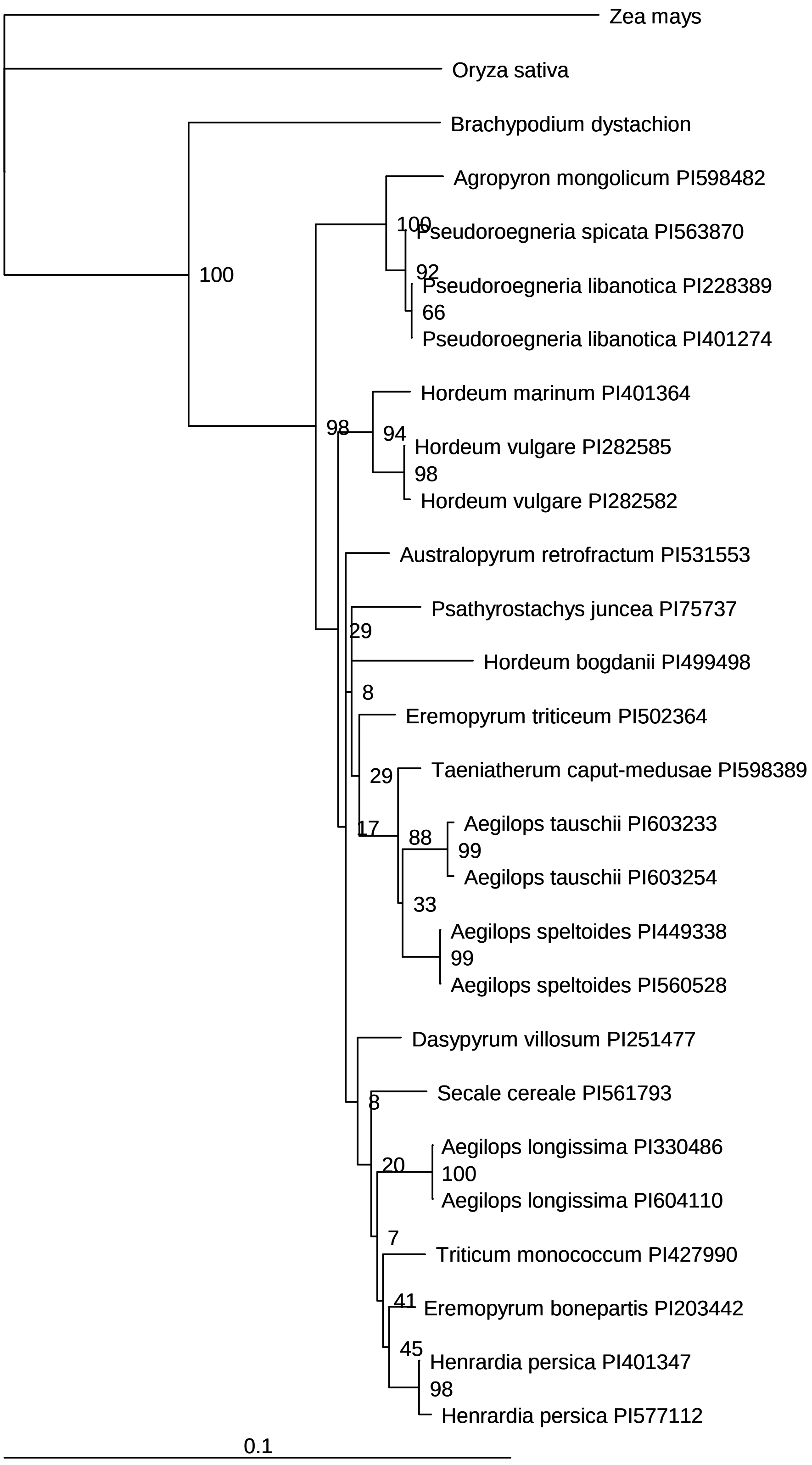

Supplement: Additional file 3 — Phylogenetic tree inferred with LOC_Os01g09300sequences. Figure S2 showing the phylogenetic tree inferred with locus LOC_Os01g09300. [file 1471-2148-11-181-S3.PDF]
