# Supplementary material for: Multigenic phylogeny and analysis of tree incongruences in Triticeae (Poaceae)
Source: BMC Evol Biol. 2011 Jun 24;11:181. doi: 10.1186/1471-2148-11-181 (PMC3142523; doi:10.1186/1471-2148-11-181)

Figure S9. Phylogenetic tree inferred with LOC_Os01g39310 sequences. Values in nodes are bootstrap values.

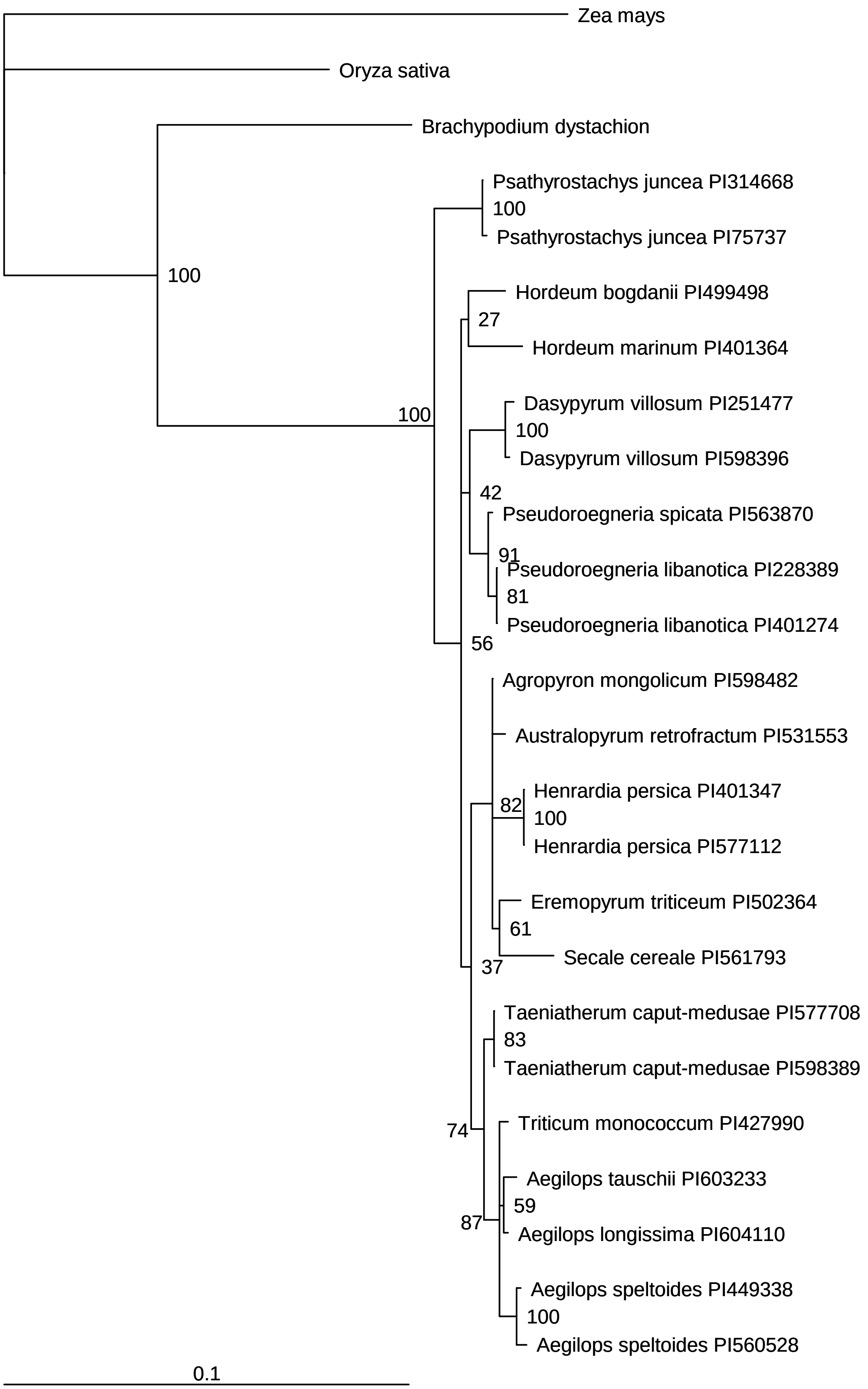

Supplement: Additional file 10 — Phylogenetic tree inferred with LOC_Os01g39310 sequences. Figure S9 showing the phylogenetic tree inferred with locus LOC_Os01g39310. [file 1471-2148-11-181-S10.PDF]
