# Supplementary material for: Multigenic phylogeny and analysis of tree incongruences in Triticeae (Poaceae)
Source: BMC Evol Biol. 2011 Jun 24;11:181. doi: 10.1186/1471-2148-11-181 (PMC3142523; doi:10.1186/1471-2148-11-181)

Figure S22. Phylogenetic tree inferred with eIFiso4E sequences. Values in nodes are bootstrap values.

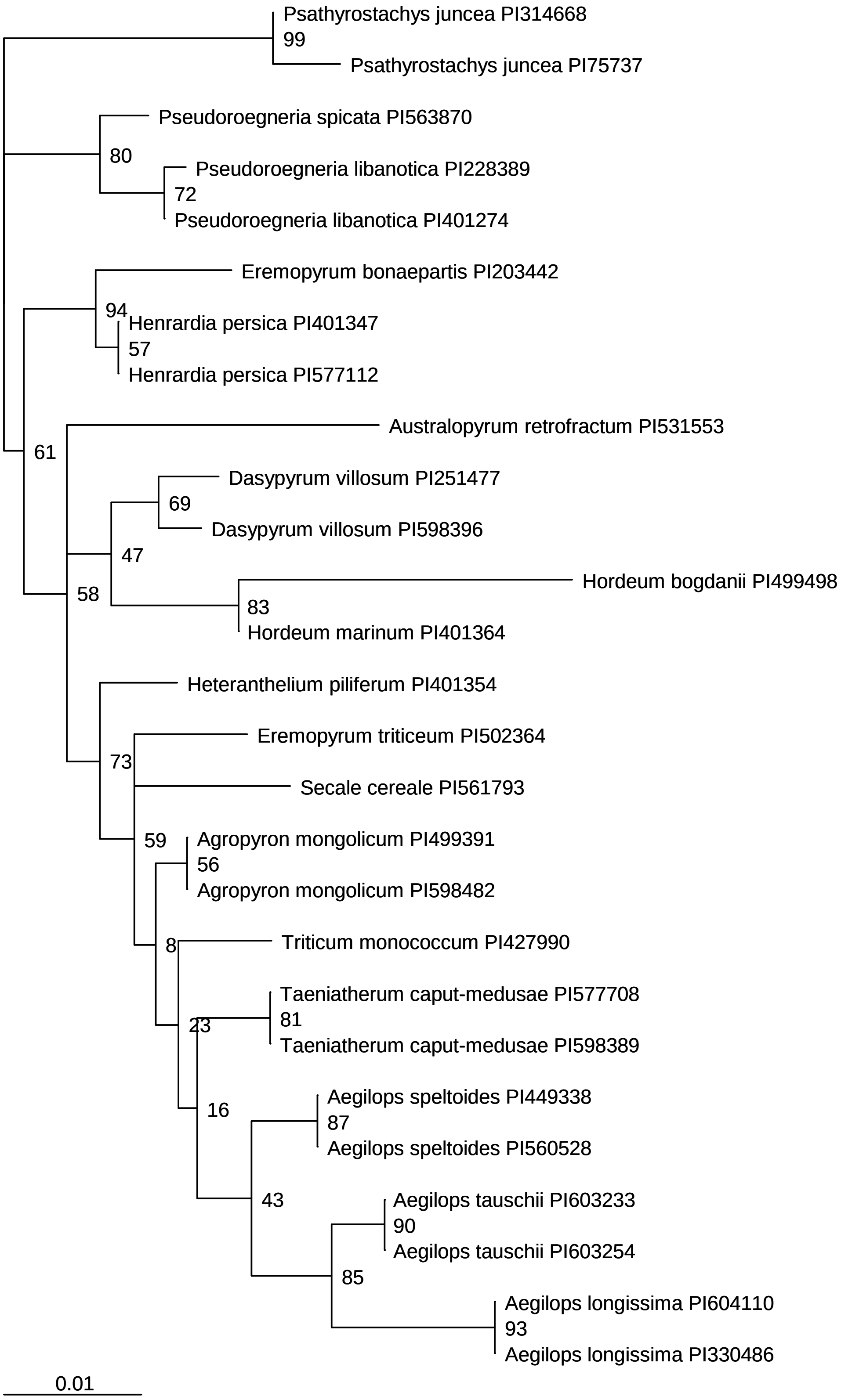

Supplement: Additional file 23 — Phylogenetic tree inferred with eIFiso4E sequences. Figure S22 showing the phylogenetic tree inferred with locus eIFiso4E. [file 1471-2148-11-181-S23.PDF]
