# Supplementary figures and images for: Multigenic phylogeny and analysis of tree incongruences in Triticeae (Poaceae)
Source: BMC Evol Biol. 2011 Jun 24;11:181. doi: 10.1186/1471-2148-11-181 (PMC3142523; doi:10.1186/1471-2148-11-181)

Figure S24. Phylogenetic tree inferred with PinA sequences. Values in nodes are bootstrap values.

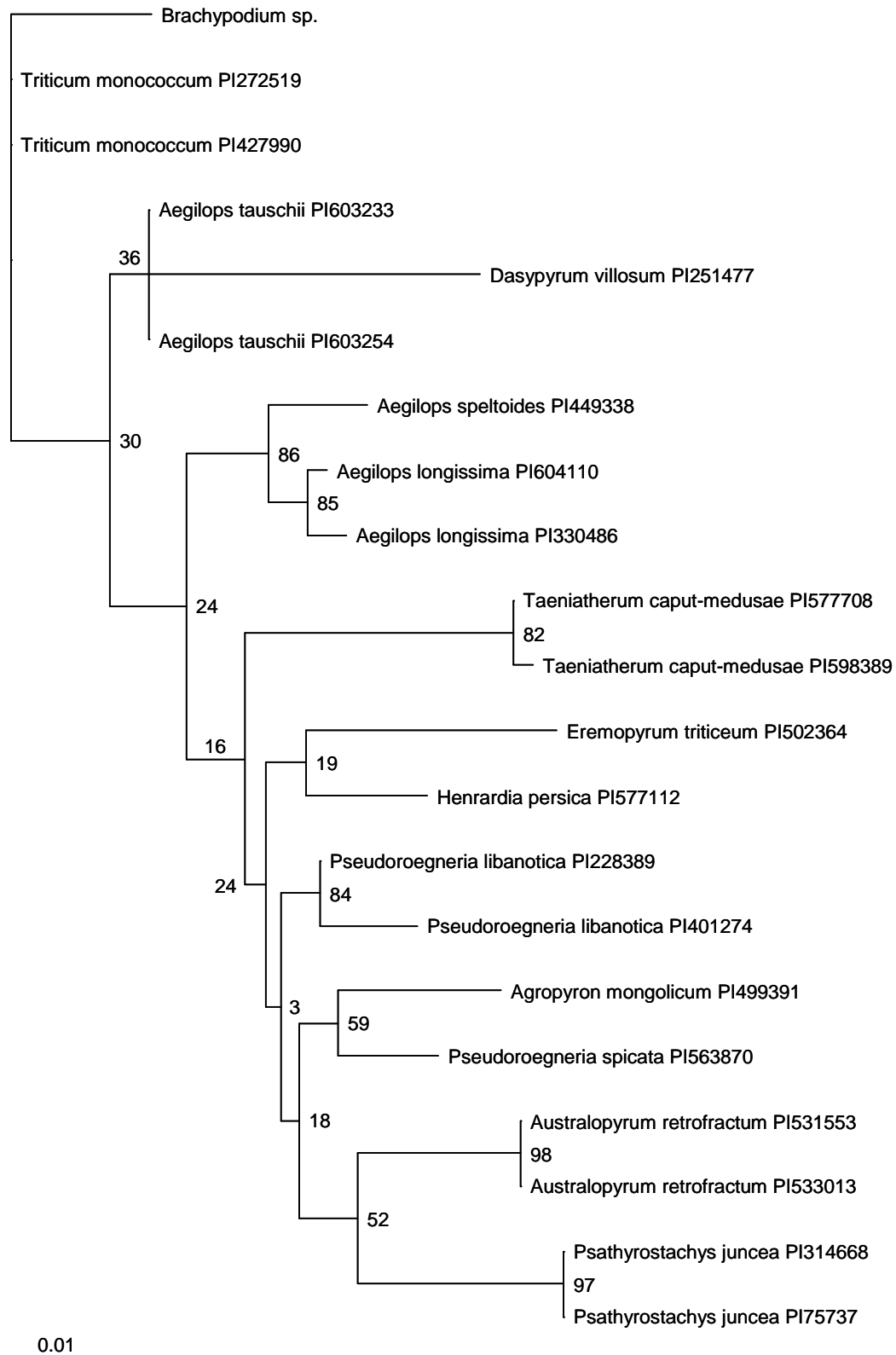

Supplement: Additional file 25 — Phylogenetic tree inferred with PinA sequences. Figure S24 showing the phylogenetic tree inferred with locus PinA. [file 1471-2148-11-181-S25.PDF]

Figure S25. Phylogenetic tree inferred with PinB sequences. Values in nodes are bootstrap values.

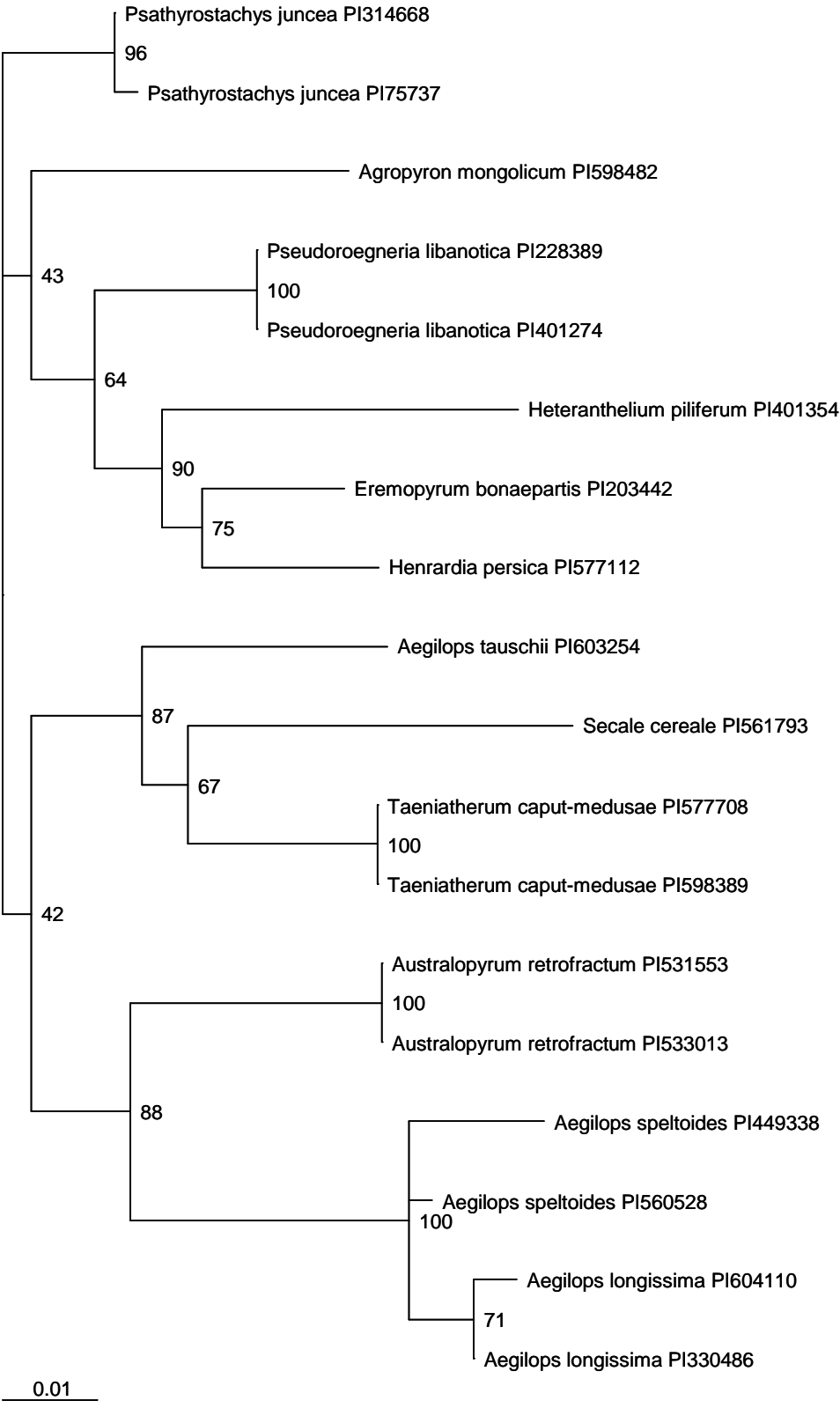

Supplement: Additional file 26 — Phylogenetic tree inferred with PinB sequences. Figure S25 showing the phylogenetic tree inferred with locus PinB. [file 1471-2148-11-181-S26.PDF]

Figure S26. Phylogenetic tree inferred with PSY2 sequences. Values in nodes are bootstrap values.

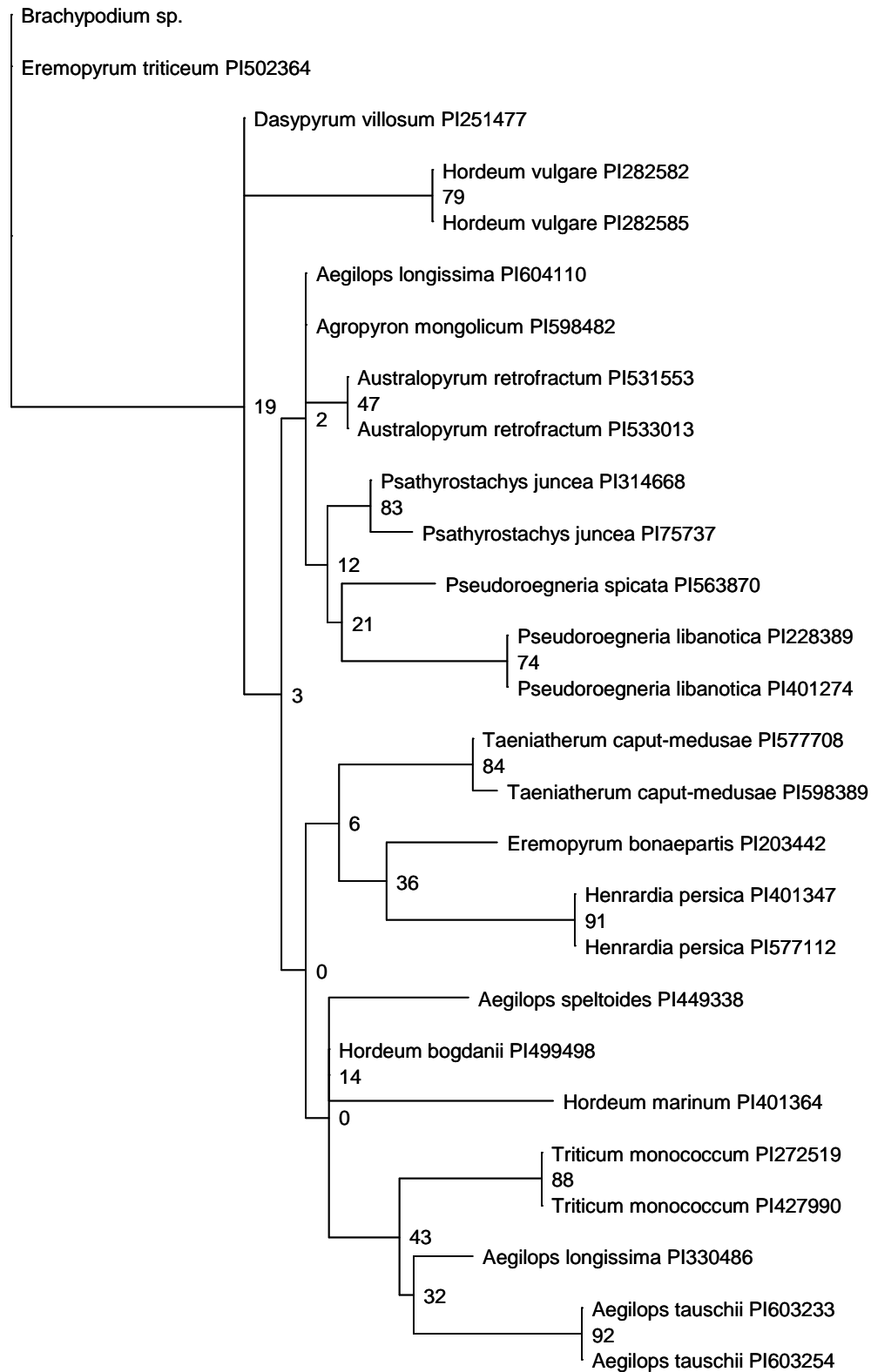

0.01

Supplement: Additional file 27 — Phylogenetic tree inferred with PSY2 sequences. Figure S26 showing the phylogenetic tree inferred with locus PSY2. [file 1471-2148-11-181-S27.PDF]

Figure S27. Phylogenetic tree inferred with MATK sequences. Values in nodes are bootstrap values.

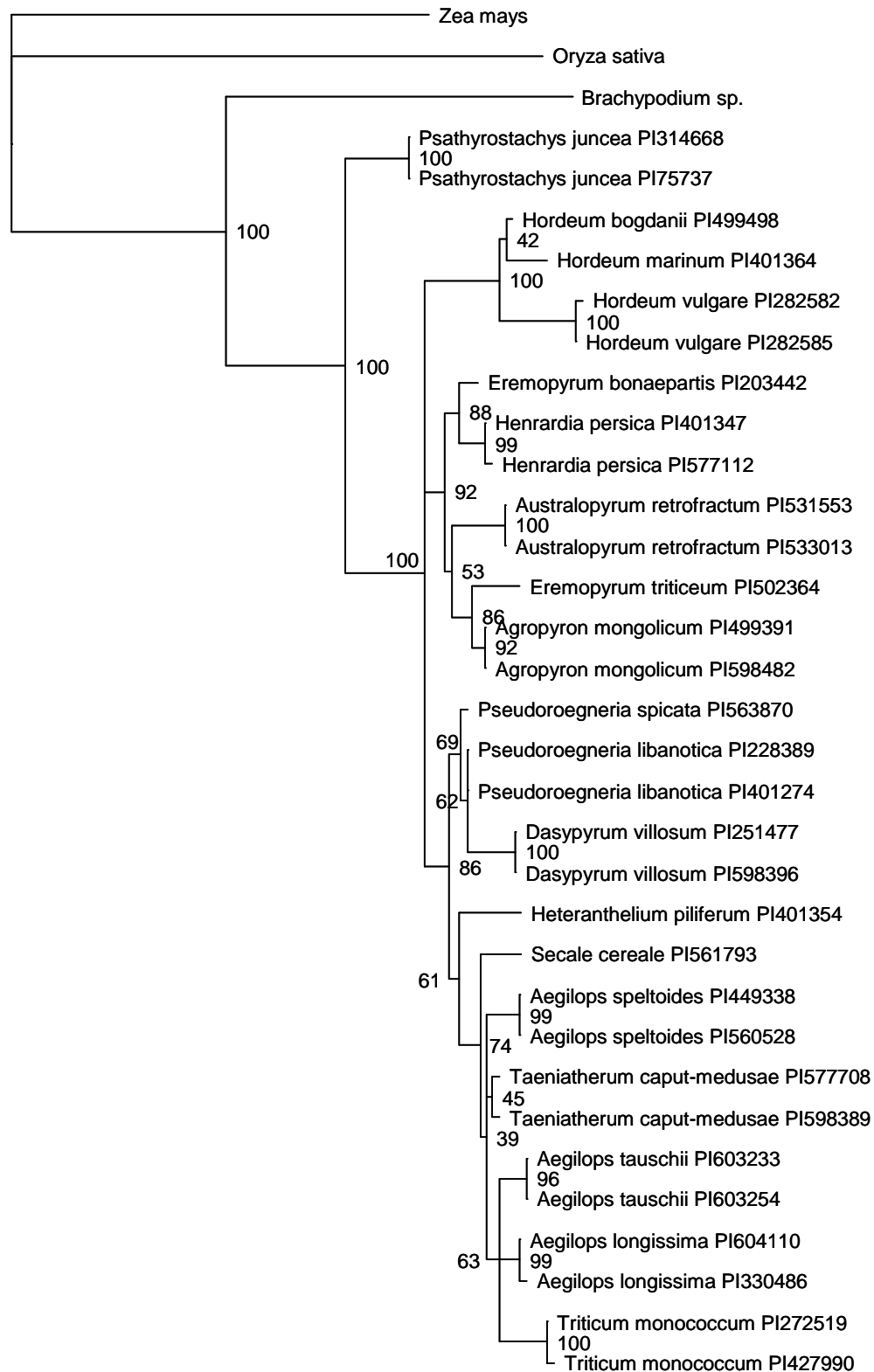

0.01

Supplement: Additional file 28 — Phylogenetic tree inferred with MATK sequences. Figure S27 showing the phylogenetic tree inferred with locus MATK. [file 1471-2148-11-181-S28.PDF]
